# Th17-driven CD8^+^ T cells in hUC-MSC and CAR T-cell dual immunotherapy for superior anti-tumor efficacy

**DOI:** 10.1038/s41419-026-08656-7

**Published:** 2026-03-27

**Authors:** Caidong Hu, Haixiao Zhang, Haojie Zhu, Jixin Fan, Dabing Chen, Shuxian Zhu, Chuo Li, Jiaqi Sun, Yifan Chen, Jinhua Ren, Xiaoming Feng, Ying Chi, Zhibo Han, Zhongchao Han, Erlie Jiang, Guanbin Zhang, Jianda Hu, Ting Yang

**Affiliations:** 1 The Second Department of Hematology, National Regional Medical Center, Binhai Campus of the First Affiliated Hospital, Fujian Medical University, Fuzhou, China; 2 The Second Department of Hematology, the First Affiliated Hospital, Fujian Medical University, Fuzhou, China; 3 Institute of Precision Medicine, Fujian Medical University, Fuzhou, China; 4 Beijing Key Laboratory of Hematopoietic Stem Cell Transplantation, National Clinical Research Center for Hematologic Disease, Peking University People’s Hospital, Peking University Institute of Hematology, Beijing, China; 5 State Key Laboratory of Experimental Hematology, National Clinical Research Center for Blood Diseases, Haihe Laboratory of Cell Ecosystem, Institute of Hematology & Blood Diseases Hospital, Chinese Academy of Medical Sciences & Peking Union Medical College, Tianjin, China; 6 Fujian Medical University, Fuzhou, China; 7 School of Intelligent Medicine, Chengdu University of Traditional Chinese Medicine, Chengdu, China; 8 Department of Laboratory Medicine, Fujian Medical University, Fuzhou, China; 9 Mianyang People’s Hospital, Mianyang, China; 10 The Second Affiliated Hospital, Fujian Medical University, Quanzhou, China

**Keywords:** Cancer immunotherapy, B-cell lymphoma

## Abstract

Chimeric antigen receptor (CAR) T-cell therapy has emerged as a promising treatment for hematological malignancies; however, its efficacy and safety remain challenging, particularly in the context of high tumor burden. High tumor load and substantial residual lesions significantly impair CAR T-cell function and exacerbate cytokine release syndrome (CRS). Here, we report the development of a novel dual cellular immunotherapy in which human umbilical cord-derived mesenchymal stem cells (hUC-MSCs) are co-administered with CD19 CAR T-cells. We demonstrated that this combination therapy enhances the anti-tumor efficacy of CD19 CAR T-cells under high tumor burden condition. In xenograft models of high tumor burden B-cell lymphoma, the dual cellular immunotherapy improved survival, mitigated myelosuppression, and preserved CAR T-cell expansion. Transcriptomic analysis of CAR T-cells revealed enrichment of the Th17 pathway in CAR T-cells, while single-cell RNA sequencing showed enhanced, particularly that of NK-like cytotoxic T lymphocytes characteristics which are associated with Th17 differentiation. Furthermore, in a CRS model, hUC-MSCs attenuate CRS severity by suppressing macrophage activity. Collectively, hUC-MSCs significantly enhance the anti-tumor capability of CD19 CAR T-cells under high tumor burden conditions by inducing CD8^+^ NK-like cytotoxic T lymphocytes through Th17 differentiation, while concurrently mitigating treatment-related side effects. Our study provides a novel therapeutic strategy to improve clinical outcomes in hematological malignancies.

## Introduction

CD19-targeted chimeric antigen receptor (CAR) T-cell therapy has revolutionized the treatment of B-cell leukemia and lymphoma, achieving remarkable remission rates of 65–90% [1, 2]. Despite the striking clinical achievements, CAR T-cell therapy is also facing several challenges associated with its efficiency and safety, especially in high tumor burden [3]. Studies have reported that increased tumor burden contributes to the poor prognosis of patients undergoing CAR T therapy [4, 5], that constant tumor stimulation causes T-cell exhaustion, which leads to decreased expansion of CAR T-cells [6, 7]. Furthermore, high tumor burden tends to create a hostile tumor microenvironment (TME) that undermines CAR T-cell expansion, anti-tumor activity, and persistence [3, 8], and is associated with increased incidence and severity of cytokine release syndrome (CRS) [9]. CRS is initiated by the activation and proliferation of CAR T-cells, which can significantly reduce their therapeutic index [10]. Moreover, treatment of CRS can also compromise the efficacy of CAR T cells, potentially affecting remission and ongoing CAR T-cell persistence. For example, Davila et al. found a dramatic decrease of 19–28z CAR T-cells in the bone marrow (BM) of CRS patients treated with high-dose steroids [11]. Recent advancements have explored various approaches to improve the efficiency and safety of CAR T-cells, including gene editing to generate more potent T-cell subsets, leveraging memory T-cell properties, and metabolic reprogramming to enhance T-cell adaptability within the TME [12–14]. However, many of these strategies target single pathways and often fail to optimize the anti-tumor efficacy and safety profile of CAR T-cell function. Therefore, new strategies to improve the efficacy and safety of CAR T-cell therapy are urgently needed.

Mesenchymal stem cells (MSCs), as a subset of stromal stem cells endowed with self- renewal capacity and multipotent differentiation potential, have been extensively utilized in the treatment of various diseases, owing to their MHC-unrestricted immunomodulatory effects, hematopoietic reconstitution capabilities, and tissue regeneration properties [15, 16]. The modulation of T cells by MSCs is bidirectional as they can exert anti-inflammatory effects by suppressing T-cell proliferation and cytokine secretion, and they can also enhance T-cell anti-tumor activity through mechanisms such as mitochondria transfer across intercellular nanotubes that improve T-cell metabolic fitness to enhance anti-tumor efficacy [17]. Nevertheless, the effects of MSCs on CAR T-cells have been poorly investigated. In a murine syngeneic model of CAR T therapy, Xia et al. found that murine MSCs had no impact on the general survival outcomes or the expansion of CAR T-cells; however, elevated platelet count and hemoglobin were found after MSCs infusion [18]. Zhang et al. found that MSCs inhibited CD19 CAR T-cell-mediated cytotoxicity in vitro, an effect dependent on stanniocalcin-1 [19]. Recent studies further demonstrated that genetically engineered MSCs can be harnessed to potentiate CAR T-cell therapy [20]. For instance, MSCs engineered to release IL-7 and IL-12 were found to promote Th1 polarization, enhance CAR T-cell expansion, reduce activation-induced cell death, and improve cytotoxic function against colorectal cancer cells [21]. Likewise, MSCs used as delivery vehicles for oncolytic adenoviruses encoding IL-12 and PD-L1 blockers can reshape the TME, markedly increasing the infiltration, polyfunctionality, and antitumor activity of HER2-specific CAR T-cells in lung cancer models [22]. These findings highlight the potential of MSCs as versatile immunomodulatory platforms to improve CAR T-cell persistence and efficacy within the tumor niche. In addition, MSCs exhibit a dual role in the TME of blood malignances [23], promote angiogenesis in the TME through the secretion of pro-angiogenic factors [24]. However, another research suggests that MSCs may exert anti-tumor effects by inducing apoptosis or differentiation of tumor cells [25]. The impact of MSCs on the TME in the context of CAR T therapy remains unclear.

Here, we aim to address the efficacy and safety of CD19 CAR T-cells in high-tumor-burden lymphoma through a dual immunotherapy approach, co-administering hUC-MSCs with CD19 CAR T-cells. Our findings indicate that hUC-MSCs promote the anti-tumor function of CAR T-cells by promoting Th17-driven differentiation of NK-like cytotoxic T lymphocytes. Moreover, hUC-MSCs effectively mitigate CRS through downregulating hyperactivated macrophages and attenuating excessive cytokine release. These findings provide a new direction for treating patients with high tumor burden and open new avenues for optimizing CAR T-cell therapy.

## Materials and Methods

### Cell lines and culture

The Raji and Nalm6 cell lines were obtained from the Institute of Hematology, Chinese Academy of Medical Sciences (Tianjin, China) and were authenticated by short tandem repeat (STR) DNA analysis. These cells were transduced with a lentivirus to stably express firefly luciferase and green fluorescent protein (GFP). Tumor cell line was cultured in RPMI-1640 medium (Gibco, CA) supplemented with 10% fetal bovine serum (FBS) (HyClone, UT), 2 mM L-glutamine (Gibco), and 100 U/mL penicillin-streptomycin (Gibco). hUC-MSCs were obtained from Tianjin Amcell Genetic Engineering Co., Ltd, and passages 4–5 were used in our study.

### Construction of CD19 CAR vector and CAR T-cell manufacturing

The recognition domain of CD19, which includes the single-chain variable fragment (scFv) from the murine anti-human CD19 antibody (clone FMC63), was fused with 4-1BB costimulatory and CD3 signaling domains and subsequently cloned into the pCDH-CMV-MCS-EF1-Puro plasmid. Peripheral blood (PB) from healthy donors was obtained from the First Affiliated Hospital of Fujian Medical University, with approval from the hospital’s ethics committee (approval number: MTCA, ECFAH of FMU [2015] 084-2). CD3^+^ T cells were isolated using an EasySep™ Human T Cell Enrichment Kit (StemCell Technologies, British Columbia, Canada), activated with the CD3/CD28 T Cell Activator (StemCell Technologies, Vancouver, Canada) for 36 hours, and transduced with lentiviral vectors carrying the CD19 CAR or the PCDH empty vector control. The CAR T-cells were cultured in T-Cell Expansion Medium (StemCell Technologies, Vancouver, Canada) supplemented with 100 ng/mL recombinant human IL-2 (Pepro Tech, New Jersey, USA) and 10% FBS. The expression of CAR was evaluated by flow cytometry on day 4, using recombinant protein L with an His Tag (Acro Biosystems, Beijing, China) and anti-His-APC antibody (BioLegend, San Diego, CA, USA). CAR T-cells were expanded in vitro until Day 7 and 10, achieving a transduction efficiency of over 40%.

### Flow cytometry detection

For surface staining, cells were incubated with monoclonal antibodies for 30 minutes at 4°C and washed with phosphate-buffered saline (PBS) containing 2% FBS. Cell viability was assessed using DAPI (SolarBio, Beijing, China), 7-AAD (BD Biosciences, CA), or Fixable Viability Dye (FVD, eBioscience, CA). All samples were analyzed using a Canto II or LSR Fortessa (BD Biosciences, CA), and data were processed with the FlowJo software (version 10.8.1; BD Biosciences, CA). Cell sorting was performed on a FACSAria II system (BD Biosciences, CA). A complete list of antibodies is provided in the Table S1.

### Cell proliferation assay

GFP-positive tumor cells (Raji) were seeded in 48-well plates at a density of 2 × 10⁵ cells per well, with or without hUC-MSCs at a density of 1 × 10⁵ cells per well, in RPMI-1640 medium. The absolute counts of tumor cells at various time points were measured using Precision Counting Beads™ (BioLegend, CA) to assess the impact of MSCs on tumor growth.

### In vitro cytotoxicity assay

hUC-MSCs (5 × 10^3^/well) were pre-incubated in 96-well plates for 4 h to ensure adhesion. GFP-positive Raji cells were mixed with CD19 CAR T or PCDH cells at different effector-to-target ratios (E: T) and co-cultured with or without hUC-MSCs in RPMI-1640 medium. CAR T cytotoxicity was evaluated over time using bead-counting flow cytometry (FCM). Specific lysis was calculated as: (1-live tumor cells in the experimental group/live tumor cells in the corresponding PCDH group) × 100%. Where indicated, the RORγt inhibitor GSK805 (10 µM, MCE, NJ, USA), purified anti-human IL-1β (1 µg/mL, BioLegend), purified anti-human IL-6 (1 µg/mL, BioLegend), recombinant human IL-1β (1 ng/mL, Pepro Tech) and recombinant human IL-6 (10 ng/mL, Pepro Tech) were added to the co-culture system [26]. Suspended cells were collected for FCM analysis of CAR-positive cells and the expression of exhaustion markers (PD-1, TIM3, LAG3) and effector markers (CD25, CD69, CD107a).

### Xenograft mouse models

CAR T-cells for in vivo experiments were purified by FCM with a CAR expression level exceeding 95%. Animal experiments were approved by the Ethics Committee of Fujian Medical University (Approval No: IACUC FJMU 2024-0161). Experiments were performed with both male and female mice unless otherwise indicated.

Using tumor-bearing immunodeficient mouse models, we assessed the in vivo antitumor activity of CAR T-cell therapy. NOD.Cg-Prkdcscid Il2rgtm1Wjl/SzJ (NSG) mice (Biocytogen, Beijing, China), aged 6–8 weeks, were injected with 3 × 10⁶ Raji-GFP-Luc cells via the tail vein. Tumor engraftment was assessed under anesthesia on day 6 using the Xenogen IVIS bioluminescence system (Caliper Life Sciences, MA). Mice were stratified based on tumor burden and received 3 × 10⁶ CD19 CAR T-cells or PCDH cells via the tail vein on day 7, followed by 3 × 10⁵ hUC-MSCs or no injection, also via the tail vein. Survival was monitored, peripheral blood counts were performed weekly using Sysmex’s flagship analyzer, and CAR T-cells (mCD45^+^hCD3^+^) and tumor cell expansion were analyzed by FCM. Tumor burden was monitored weekly. Mice were euthanized if they experienced > 20% weight loss or severe debilitation/paralysis.

An immunodeficient mouse model retaining macrophage activity was developed to study CRS. High-dose CAR-T administration following high tumor burden establishment induced rapid tumor lysis and consequent cytokine storm [27]. CB17.Cg-PrkdcscidLystbg-J/Crl (SCID-beige) mice, aged 6-8 weeks (Viton Lihua Experimental Animal Technology, Beijing, China), were injected intraperitoneally with 3 × 10⁶ Raji-GFP-Luc cells. Tumor burden was quantified by bioluminescence imaging (Xenogen IVIS) on day 20. For the high tumor burden model, mice received an intraperitoneal injection of 3 × 10⁷ CD19 CAR T-cells on day 21. In the hUC-MSC pretreatment group, 3 × 10⁶ hUC-MSCs were administered intraperitoneally 12 h prior to CAR T-cell injection. Based on clinical experience, mice were treated with a murine IL-6R blocking antibody (clone 15A7, BioXcell, West Lebanon, NH, USA) as positive control. In the anti-mIL-6R group, 50 μg anti-mIL-6R was administered intraperitoneally 4 h before CAR T injection, followed by 25 μg anti-mIL-6R at 24 and 48 hours. Survival and weight were monitored daily. Tail vein blood was collected before or 48 h after CAR T-cells injection, allowed to clot for 30 min, and centrifuged at 3000 rpm for 20 min. The supernatant (serum) was stored at −80 °C. Additional tail blood samples were collected at 12, 24, 48, and 72 h, and spleen and BM cells were harvested at 72 h for FCM.

### Immunohistochemistry

Organs from SCID-beige mice were collected 72 h post CAR T-cells injection, fixed in paraformaldehyde for 48 h, and paraffin-embedded. Immunohistochemistry was performed on 5-μm sections. Citric acid (pH 6.0) was used to repair the antigen by microwaving for 8 min on medium heat, stopped heating for 8 min and then heat again on medium-low for 7 minutes. Neutrophils were labeled with anti-mouse Ly6G (Servicebio, Wuhan, China) at a dilution of 1: 1000, and macrophages were labeled with anti-mouse F4/80 (Servicebio) at a dilution of 1: 1000. The primary antibodies were incubated at room temperature for 60 minutes and then incubated at room temperature for 20 minutes with horseradish peroxidase-conjugated goat anti-rabbit IgG secondary antibody. Nuclei were stained with DAPI (SolarBio) at a dilution of 1: 1000 for 5 min. Images were acquired with an Olympus BX43F microscope and analyzed using Aipathwell software (Servicebio). Three regions per sample were analyzed to calculate the average positive cell density, defined as positive cells per unit area (*n* = 3).

### Cytokine measurements

The LEGENDplexTM Human Essential Immune Response Panel (BioLegend) was used to measure cytokines in the co-culture supernatants. Data were analyzed on the online platform (https://legendplex.qognit.com). Serum cytokines were measured using Bio-Plex Pro mouse cytokine assays (Bio-Rad, CA) per the manufacturer’s instructions. Cytokine fold changes were calculated using the formula: N48 = N0 × 2^n^, where N0 is the baseline cytokine level, N48 is the 48th h cytokine level, and n represents the fold change.

### RNA extraction and real-time quantitative reverse transcription PCR (RT-qPCR)

Total RNA was extracted using the TRIzol reagent (Thermo Fisher Scientific, Waltham, USA), followed by cDNA preparation using a reverse transcription reagent kit (Promega Corporation, Madison, USA). RT-qPCR was performed using the SYBR Green Supermix (Vazyme Biotechnology, Shanghai, China) on an Applied Biosystems 7500 Real-Time PCR System. All primer sequences are listed in Table S2.

### RNA-Sequencing analysis

RNA sequencing was performed using the Illumina NovaSeq-PE150 system (BerryGenomics and Novogene, Tianjin, China). CD19 CAR T-cells were isolated by flow cytometry based on the expression of DAPI^-^GFP^-^ CAR^+^ after 48 h of co-culture with Raji cells at a 1:5 ratio, while the mCAR-T group samples were collected from the 48 h co-culture system containing hUC-MSC, Raji cells and MSCs at a 10:50:10 ratio. Raw data in the fastq format were first processed and quality controlled by Fast QC. Clean data were then obtained by removing reads containing adapters. The DeSeq2 R package was used for differential gene analysis [28]. GO and KEGG enrichment analyses were conducted using the NovaMajic platform (https://magic-plus.novogene.com/) [29]. GSEA enrichment was performed using the GSEA software (Broad Institute). Gene sets from the Molecular Signature Database (GSEA|MsigDB) (gsea-msigdb.org) included: TNF SIGNALING PATHWAY (HSA04668), IL-17 SIGNALING PATHWAYS. Furthermore, custom gene sets also be used: CYTOLYTICS T EFFECTOR PATHWAY (*EOMES, TBX21, GZMB, PRF1, FASL, GZMH, GZMA*); CYTOLYTICS T (*CX3CR1, PRF1, GZMA, GZMB, GZMH, GNLY, FGFBP2, KLRG1, FCGR3A, GZMK, LYAR, GZMM, TXNIP, FCRL6, NKG7, KLRD1*); CHEMOKINE (*CSF1, CSF2, CSF3, CXCL8, HLA-DRA, HLA-DRB1, HLA-DRB3, HLA-DRB4, HLA-DRB5, IFNA1, IFNB1, IFNG, IL10, IL11, IL13, IL15, IL1A, IL2, IL3, IL4, IL5, IL6, IL7, PDGFA, TGFB1, TGFB2, TGFB3, TNF*).

### Single-cell sorting and processing of 10× Genomics single-cell RNA sequencing

Based on FACS analysis, DAPI⁻GFP^-^CAR⁺ cells were cultured identically to bulk transcriptome conditions before sorting. The sorted populations were resuspended in FACS buffer (phosphate-buffered saline with 0.04% BSA) and immediately processed for single-cell RNA-seq library preparation.

### Single-cell RNA library construction and sequencing

In accordance with the manufacturer’s guidelines, single-cell RNA-seq libraries were constructed using the 10× Chromium Single Cell 5’ Platform (10× Genomics, Pleasanton, CA, USA). To generate single-cell gel beads in emulsion (GEMs), protoplasts were briefly loaded onto a chromium microfluidic chip. The single-cell 5ʹ gene expression libraries were generated using the Chromium Next GEM Single Cell 5’ Kit v2 (10× Genomics, Pleasanton, CA, USA). Subsequently, the Agilent 2100 Bioanalyzer system was used to assess the quality of the libraries, and the Illumina NovaSeq 6000 platform (Illumina, San Diego, CA, USA) was used for sequencing the data.

### Single-cell RNA sequencing analysis

CellRanger v7.1.0 software (https://www.10xgenomics.com/support/cn/software/cell-ranger/downloads/previous-versions) was used to align the sequencing data for each sample to the reconstructed human reference genome, facilitating the cell identification and expression matrix construction. Quality control was performed on the expression matrices of each sample using the Seurat v5.0.3 software (https://satijalab.org/seurat). Quality control criteria were as follows: 1) cells expressing a minimum of 200 genes and a maximum of 7000 genes were retained; 2) cells with mitochondrial gene expression less than 5% and more than 0.5% were retained; and 3) genes detectable in at least 3 cells were retained. The DoubletFinder v2.0.4 package (https://github.com/chris-mcginnis-ucsf/DoubletFinder) was used to eliminate doublets (two or more cells in a single oil droplet). The harmony algorithm implemented in the Seurat software was used to integrate two sample datasets. The integrated data assay was then scaled in Seurat using the ScaleData function. Principal component analysis (PCA) was performed on the top 2000 highly variable genes to reduce the linear dimensionality. The first 50 principal components (PCs) were used in the Seurat function FindNeighbors and FindClusters, employing a resolution of 0.6 to identify cell clusters. The resulting cell clusters were visualized and investigated using UMAP. Cluster-enriched genes were identified using the Seurat function FindAllMarkers, with a minimum percentage (min.pct) set at 0.25.

### Single-cell GSEA analysis

GSEA analysis was performed using the fgsea package (v1.26.0). The Hallmark gene set databases from MSigDB (v7.5.1) were tested for subgroup. The gene set included:

GSE28726_NAIVE_CD4_TCELL_VS_NAIVE_VA24NEG_NKTCELL_UP;

GOLDRATH_EFF_VS_MEMORY_CD8_TCELL_UP;

GOLDRATH_NAIVE_VS_EFF_CD8_TCELL_DN;

TRAVAGLINI_LUNG_PROLIFERATING_NK_T_CEL;

TRAVAGLINI_LUNG_CD8_NAIVE_T_CELL;

TRAVAGLINI_LUNG_CD8_NAIVE_T_CELLHA;

NG_CD8_NAIVE_T_CELLHAY_BON;

KAECH_DAY8_EFF_VS_MEMORY_CD8_TCELL_UP;

HAY_BONE_MARROW_CD8_T_CELL;

KAECH_DAY8_EFF_VS_DAY15_EFF_CD8_TCELL_UP;

DURANTE_ADULT_OLFACTORY_NEUROEPITHELIUM_NK_CELLS;

HADDAD_T_LYMPHOCYTE_AND_NK_PROGENITOR_DN;

WP_PI3KAKTMTOR_VITD3_SIGNALING;

GSE39110_DAY3_VS_DAY6_POST_IMMUNIZATION_CD8_TCELL_DN;

GSE15750_DAY6_VS_DAY10_TRAF6KO_EFF_CD8_TCELL_UP;

GSE15750_DAY6_VS_DAY10_EFF_CD8_TCELL_UP.

### Gene set scoring

The AUCell algorithm was used for the quantification of gene set signatures in each cell [30]. NK characterized gene set: *CD160*, *CD244*, *CHST12*, *CST7*, *GNLY*, *IL18RAP*, *IL2RB*, *KLRC1*, *KLRC3*, *KLRD1*, *KLRF1*, *PRF1*, and *XCL2* [31]. Exhaustion characterized gene set: *LAG3, TIGIT, PDCD1, CTLA4, HAVCR2, TOX, PRDM1, and MAF* [32]. UMAP plot representation was used to assess the distribution of module scores for NK cell; Violin plots representation was used to assess the level of module scores for NK and T cell exhaustion, grouped by subset.

### Statistical analysis

All in vitro experiments were conducted in triplicate or more and analyzed using GraphPad Prism (version 8.0.0; GraphPad Software, MA). Data are presented in graphs as mean ± SD or as individual values where appropriate and all data are representative of at least three independent experiments. Statistical comparisons were performed using a two-sided unpaired *t*-test for two groups and a one-way ANOVA for more than two groups. Survival analysis of mice was conducted using the log-rank (Mantel-Cox) test. Statistical significance was defined as *P* < 0.05.

## Results

### Dual cellular immunotherapy using hUC-MSCs and CD19 CAR T-cells boosts CAR T-cell cytotoxicity and anti-tumor efficacy in high tumor burden models

**Fig. 1:**
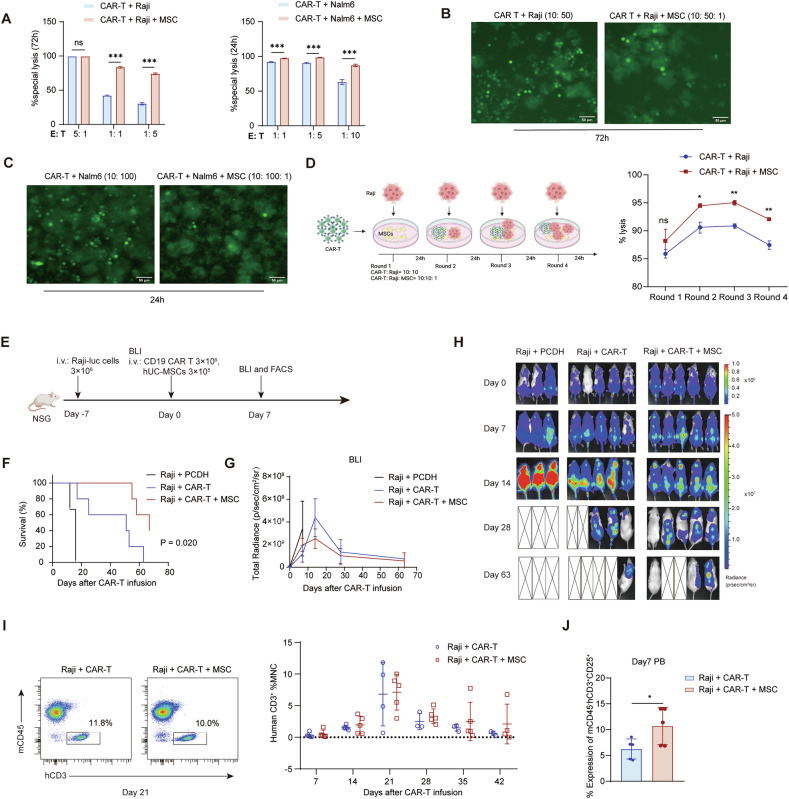
Dual cellular immunotherapy with MSCs and CD19 CAR T-cells boosts cytotoxicity and anti-tumor efficacy in high tumor burden models. **A** The specific cytotoxic activity of CAR T-cells was measured using bead-counting FCM. CAR T-cells and hUC-MSCs were co-cultured at a fixed 10:1 ratio, while tumor cells were seeded at varying effector-to-target ratios (E: T) (*n* = 3); **B**,**C** Fluorescence microscopy images showing cytotoxicity, with green fluorescent cells indicating viable GFP^+^-Raji or GFP^+^-Nalm6 cells; **D** Experimental design and timepoint-specific cytotoxicity assessment in multiple rounds of tumor challenge assays; **E** Diagram of the in vivo experiment; **F** Kaplan-Meier survival analysis shows a significant survival benefit for mice treated with CAR T-cells co-administrated with hUC-MSCs compared to controls (*n* = 3 or 5); **G**,**H** Tumor growth monitored by bioluminescence (BLI) at the indicated time points; **I** CD19 CAR T-cell expansion was evaluated by measuring the percentage of human CD3 positive and mouse CD45 negative cells in the peripheral blood (PB) at multiple time points. (MNC: Mononuclear Cells); **J** Expression of CD25 on mCD45^-^hCD3^+^ cells. Statistical significance between treatment groups was determined using a two-tailed unpaired t-test. *p < 0.05; **p < 0.01; ***p < 0.001.

MSCs have been shown to be effective regulators of T-cell function and differentiation [33]. To investigate the interaction between MSCs and CD19 CAR T-cells, we established a co-culture system where tumor cells are cultured with MSCs and CAR T-cells. hUC-MSCs, as the most clinically used type due to their abundant supply, ethical acceptability, and proven safety, and the Raji cell line (CD19^+^), a human B-cell lymphoma, were used. CAR T-cells were successfully prepared by transducing human peripheral blood mononuclear cells (PBMCs) with a lentivirus encoding an anti-CD19 scFv and 4-1BB/CD3ζ CAR (Fig. S1A, B). First, we confirmed that hUC-MSCs had no significant effect on the proliferation of Raji cells in vitro (Fig. S1C); then, we performed an in vitro killing assay at various E: T ratios (range from 10: 2 to 10: 50) [34] to evaluate the effect of hUC-MSCs on the cytotoxic efficacy of CD19 CAR T-cells against Raji cells, and calculated the specific lysis ratio. hUC-MSCs enhanced the cytotoxic activity of CAR T-cells against Raji cells at E:T ratios of 1: 1 and 1: 5, and further potentiated their killing efficacy against Nalm6 cells at E:T ratios of 1: 1, 1: 5, and 1: 10 (Fig. 1A–C). Notably, this enhancement was more pronounced at lower E:T ratios (indicating higher tumor burden). These results demonstrate that MSCs significantly boost the anti-tumor capacity of CD19 CAR T-cells, particularly under high tumor burden conditions. The hypothesis was further validated through multiple rounds of tumor challenge, which revealed that hUC-MSCs co-administered CAR T-cells maintained enhanced cytotoxic capacity upon re-exposure to tumor cells (Fig. 1D).

As co-administration of CD19 CAR T-cells with hUC-MSCs resulted in improved cytotoxicity at low E:T ratio in vitro, a high tumor burden mouse model of Raji cell-based non-Hodgkin’s lymphoma (NHL) was established according to a previous report [35] (Fig. 1E). We first rigorously excluded the potential impact of MSCs on in vivo expansion of Raji cells (Fig. S1D–F). CAR T-cell efficacy was assessed by tracking tumor progression, monitoring T-cell expansion weekly and survival. CAR T-cell safety was assessed by weekly monitoring PB cell counts. Compared to the group treated with CAR T-cells alone (CAR-T), the group treated with CD19 CAR T-cells and hUC-MSCs (mCAR-T) significantly improved mice survival (*p* < 0.05, Fig. 1F) and showed better tumor elimination (Fig. 1G, H). Although no significant difference in the expansion of hCD3^+^mCD45^+^ cells was detected in PB (Fig. 1I), we notably found higher expression levels of the activation marker CD25 on CAR T-cells when co-administered with hUC-MSCs (*p* < 0.05, Fig. 1J). Cytopenia is a common side effect of CAR T-cell therapy and hUC-MSCs are a promising strategy to address it [18]. In line with Xia et al.‘s report on MSC-mediated enhancement of BM recovery following CAR T therapy, hUC-MSCs unexpectedly mitigated the significant reduction of platelet counts after CAR T-cell infusion, which was marked in the CAR-T group (Fig. S1G). We also used a low tumor burden mice model (5 × 10^5^ Raji cells), treated with CAR T-cells +/- hUC-MSCs (Fig. S1H). Interestingly, in this low tumor burden model, CAR T-cells + hUC-MSCs showed no significant advantage in tumor elimination and survival (Fig. S1I–K), and expansion of CAR T-cells (Fig. S1L). hUC-MSC-mediated enhancement of CAR-T anti-tumor efficacy was further validated in a high-tumor-burden Nalm6 model, demonstrated by prolonged survival and reduced tumor burden in peripheral blood of MSC-treated mice (Fig. S2A–F). These results demonstrate that hUC-MSCs enhance CD19 CAR T-cell efficacy against high tumor burden, while alleviating therapy-related cytopenia in vivo.

### Dual cellular immunotherapy using hUC-MSCs and CD19 CAR T-Cells mediates stronger transcriptional signatures associated with effector functions

**Fig. 2:**
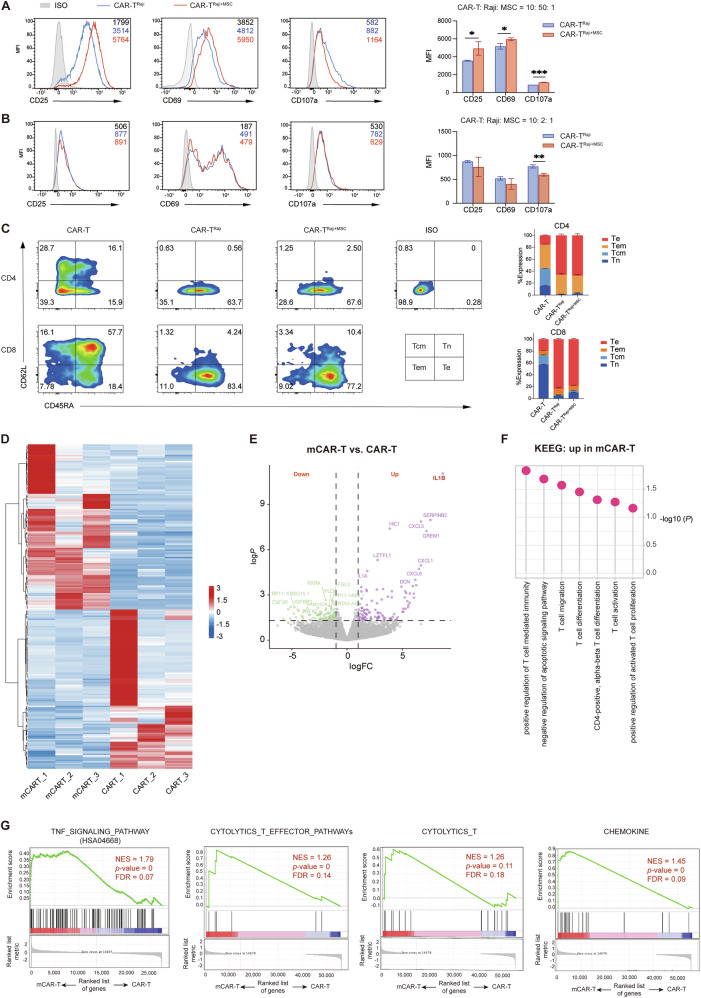
Dual cellular immunotherapy with MSCs and CD19 CAR T-Cells mediates stronger transcriptional signatures associated with effector functions. **A** CD19 CAR T-cell activation markers (CD25, CD69, CD107a) at a E: T ratio of 1: 5 and representative flow cytometry. The combination of CAR T-cells and MSCs significantly upregulated the expression of T-cell activation markers (*n* = 3). **B** Activation markers (CD25, CD69, CD107a) of CAR-T at a E: T ratio of 5: 1 and representative flow cytometry. The CAR-T/MSC combination showed no significant change in activation marker expression (*n* = 3). **C** Phenotypes of CAR T-cells determined by CD62L and CD45RA expression under a E:T ratio of 1:5. (Tn: Naïve T cells; Tcm: Central Memory T cells; Tem: Effector Memory T cells; Te: Effector T cells.) CD19 CAR T-cells from three different healthy donors were co-cultured with Raji cells for 48 h in the presence of hUC-MSCs or not, followed by sorting for both bulk and single-cell RNA sequencing (RNA-Seq). **D**,**E** Hierarchical clustering and volcano analysis of the RNA-seq results reveal differentially expressed genes between mCAR-T and CAR-T (*n* = 3); **F** Significantly upregulated gene sets in the mCAR T-cells analyzed by KEGG (*n* = 3); **G** Representative GSEA enrichment plot demonstrating the upregulation of cytolysis-related genes in mCAR T-cells (*n* = 3); *P* values were calculated by two-tailed unpaired *t*-tests. *p < 0.05; **p < 0.01.

To figure out the time-dependent and tumor burden-related effects in CAR T-cell killing assay with hUC-MSCs, we assessed the effect and exhaustion immunophenotypes of CAR T-cells under different E:T ratio. FCM analysis revealed that mCAR group under the low E: T ratio of 1: 5 showed a significant upregulation of activation markers (CD25, CD69 and CD107a) while not significant at a E:T ratio of 5:1 (Fig. 2A, B). The expression of exhaustion markers (PD-1, LAG3 and TIM3) did not differ regardless the E: T ratio (Fig. S3A, B). T-cell subpopulations were evaluated after 48 hours of co-culture at a low effector-to-target (E: T) ratio and the CD62L⁺CD45RA⁺ CAR T-cells, a subset associated with enhanced stem-like properties and sustained proliferative capacity, were increased in mCAR group (Fig. 2C). These findings may account for the more robust enhancement of CAR T-cell-mediated tumor killing by hUC-MSCs in high tumor burden settings.

To investigate the enhancement of CAR T-cell cytotoxicity by hUC-MSCs under high tumor burden conditions, we analyzed the transcriptomic profiles of CAR T-cells derived from three different donors, co-cultured with Raji cells ± hUC-MSCs (mCAR-T vs. CAR-T) at an E: T ratio of 1: 5. As expected, differences in the transcriptional profiles were observed between the 2 groups, with 123 genes upregulated in the mCAR-T group (|log2FC | ≥ 1, *p* value ≤ 0.05, Fig. 2D, E). Kyoto Encyclopedia of Genes and Genomes (KEGG) analysis revealed that the differently enriched genes in the mCAR-T group belonged to signaling pathways related to T-cell activation, proliferation, cytolysis, differentiation and chemokine activity (Fig. 2F). Gene Set Enrichment Analysis (GSEA) revealed a significant gene enrichment in the TNF signaling pathway in the mCAR-T group, and showed cytolysis, effector and chemokine gene signatures enriched in the mCAR-T group, consistent with the KEEG analysis (Fig. 2G). These findings suggest that CAR T-cells in mCAR-T group exhibit an enhanced functional phenotype, which may contribute to their stronger antitumor efficacy.

### CD8^+^ NK-like CTLs mediate superior anti-tumor effects in dual cellular immunotherapy

**Fig. 3:**
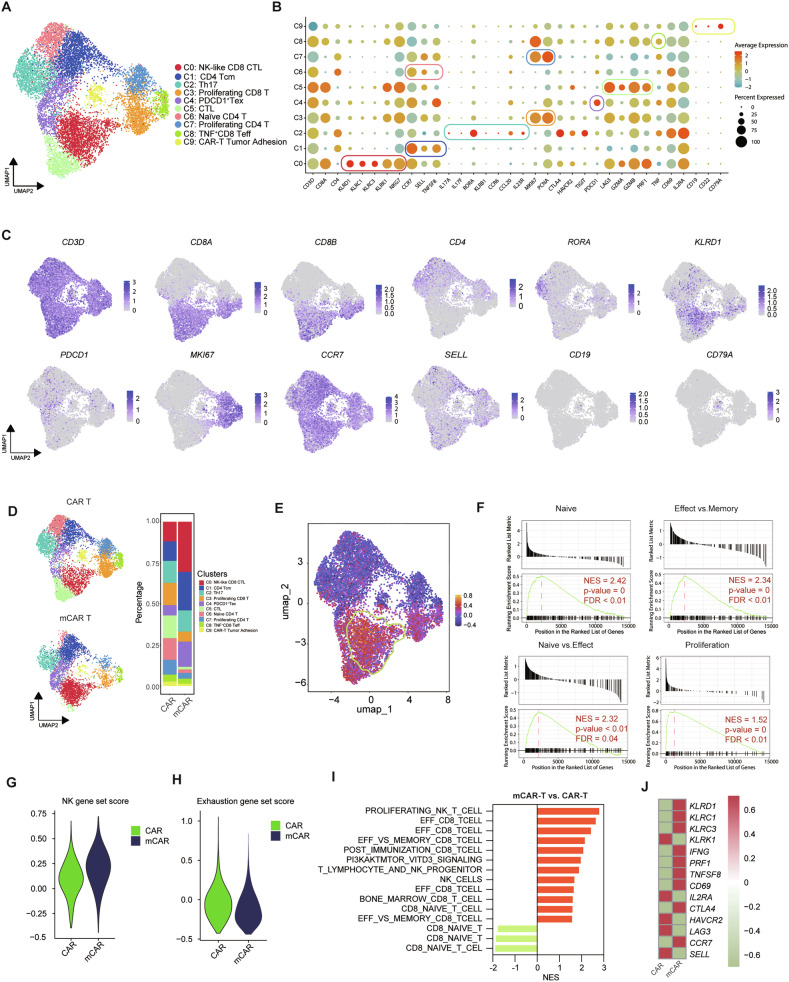
CD8^+^ NK-Like CTLs mediate superior anti-tumor effects following dual cellular immunotherapy. **A** UMAP visualization of 10272 cells from the CAR and mCAR groups. The ten clusters are indicated by different colors; **B** Dot plot illustrating the expression of marker genes in different clusters; **C** Expression of selected genes overlaid onto the UMAP plots; **D** UMAP visualization of cells in each group (right) and relative percentage of T-cell clusters from CAR and mCAR group; **E** NK scores mapped to the UMAP plot. The green circle indicates the highest score area; **F** GSEA analysis reveals enrichment for pathways associated with naïve, effector, and proliferation in the CD8^+^ clusters (Cluster 0, 3, 5, and 8) of mCAR group, as compared with CAR group; **G**,**H** Violin plots show NK scores and exhaustion scores of NK-like CTLs (Cluster 0) in the two groups; **I** GSEA scores of T cell- and NK cell-related pathways for NK-like CTL (Cluster 0) in these two groups. **J** Heatmap displays the expression of selected genes.

To better elucidate the impact of hUC-MSCs on CAR T-cell transcriptome, we performed single-cell transcriptome analysis on flow-sorted DAPI^-^CD3^+^CAR^+^ T cells, which were obtained from a 48 h killing assay with Raji cells at E:T = 1:5. After quality control, 6247 cells from the CAR-T group and 4025 cells from the mCAR-T group were successfully analyzed. Following uniform manifold approximation and projection (UMAP) analysis, there was no obvious batch effect between the two samples (Fig. S4A). Ten distinct clusters were identified and visualized by UMAP, as shown in Fig. 3A–C. Single-cell differential gene expression analysis among the ten clusters revealed notable differences in molecular profiles and the top 10 genes of each cluster were shown as heatmap (Fig. S4B). Those included four CD4^+^ clusters (Cluster 1, 2, 6 and 7), four CD8^+^ clusters (Cluster 0, 3, 5 and 8), one T exhaustion (Tex) cluster (Cluster 4) and one other undefined cluster (Cluster 9). Cluster 2 had high expression of Th17 marker genes (*IL17A*, *IL17F*, *RORA*, *KLRB1*, *CCR6*, *CCL20*, and *IL23R*), with RORA being particularly prominent, and was identified as Th17 cells. Cluster 0, characterized by high expression of *CD8A*, *CD8B*, NK-related marker genes (*KLRD1*, *KLRC1*, *KLRC3*, *KLRK1*, and *NKG7*) and effector genes (*GZMA*, *GZMB*, and *PRF1*), was identified as NK-like cytolytic T-cells (CTL) (Fig. 3B, C). Both Cluster 1 and 6 highly expressed *CCR7* and *SELL* and had low expression of activation, effector and exhaustion markers. *TNFSF8* was used to separate these two clusters into central memory T (Tcm) and naïve T-cells. Cluster 3 and 7 with highly expressed *MKI67* and *PCNA* were defined as proliferating CD8 and CD4 T cells, respectively. Cluster 9, with B cell-based tumor markers (*CD19, CD22* and *CD79A*) and T cell marker (*CD3D*) was defined as CAR T-cells adhered to tumor cells (Fig. 3A–C).

Cell subpopulations and their proportions vary between the CAR-T and mCAR-T groups. Component proportion analysis revealed that the mCAR-T group had a slightly lower proportion of CD8^+^ cluster cells (40.57 vs. 43.57%) and a lower proportion of CD4^+^ cluster cells (41.69 vs. 47.5%). Although PDCD1^+^ Tex increased in the mCAR group, we also observed an increase of CD4^+^ Tcm in the mCAR-T group (Fig. 3D), which has been associated with long-term remission in a previous report [36].

To validate the role of hUC-MSCs on CD8^+^ T cells, we compared CD8^+^ T subsets (Cluster 0, 3, 5, and 8) between the CAR-T group and the mCAR-T group. Although there was comparable percentage of CD8^+^ T cells between the 2 groups, CTL (Cluster 5) and proliferating CD8^+^ T-cells (Cluster 3) were strongly reduced in the mCAR-T group; on the contrary, NK-like CTLs (Cluster 0) dramatically increased in the mCAR-T group, suggesting that hUC-MSCs may increase NK-like CTLs (Fig. 3E). Functionally, GSEA analysis on CD8^+^ T cells from two these groups revealed an enrichment of the naïve, effector and proliferation pathways in the mCAR-T group (Fig. 3F). Next, we compared the NK score of NK-like CTLs (Cluster 0) between the CAR-T and mCAR-T groups and showed an increased NK score of NK-like CTLs in the mCAR-T group (Fig. 3G). An exhaustion gene set (*LAG3, TIGIT, PDCD1, CTLA4, HAVCR2, TOX, PRDM1*, and *MAF*) [32] was used in Cluster 0, and showed that NK-like CTLs in the mCAR-T group were not characterized by high exhaustion (Fig. 3H), unlike data from previous reports [37]. GSEA analysis of Cluster 0 revealed the upregulation of CD8 effector and NK-related pathways and downregulation of CD8 naïve-related pathways in the mCAR-T group (Fig. 3I). Specifically, NK-like CTLs had a greater association with NK-related (*KLRD1, KLRC3, KLRC1* and *KLRK1*), T cell activation (*CD69, IL2RA*, and *IL2*) and effector function (*IFN* and *PRF1*) genes (Fig. 3J). These data suggest that dual cellular immunotherapy upregulates an NK-like CD8^+^ CTL subset with enhanced cytotoxicity and reduced exhaustion.

### Dual cellular immunotherapy upregulates the Th17 pathway

**Fig. 4:**
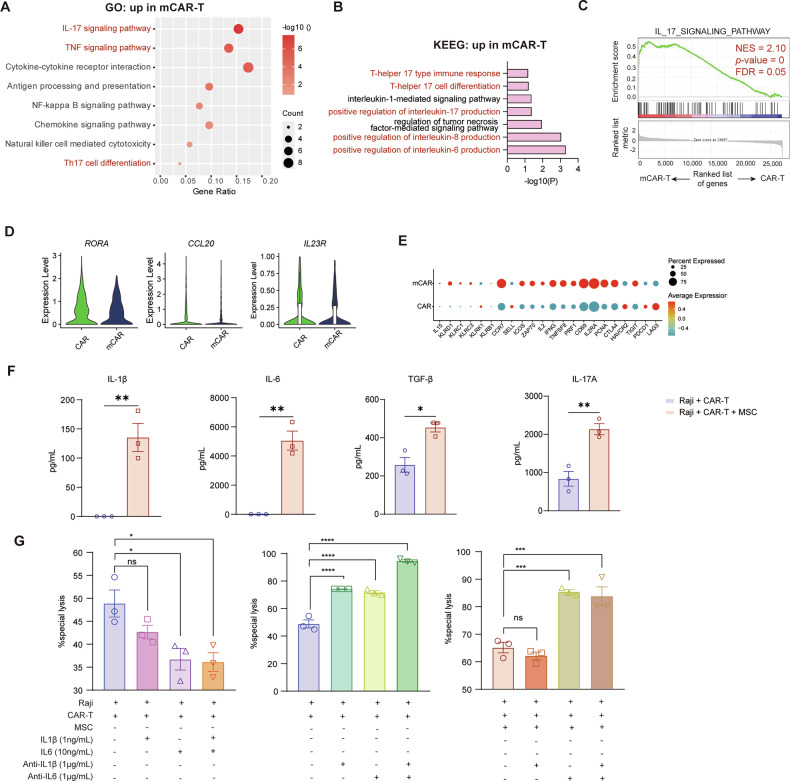
Dual cellular immunotherapy upregulates the Th17 pathway. **A**-**C** Enrichment analysis of MSC-modulated transcriptional profiles in CAR T-cells derived from three distinct healthy donors. Significantly upregulated gene sets related to IL-17 and Th17 signaling pathway in GO, KEGG and GSEA. (*n* = 3); **D** Violin plots show the expression levels of the Th17 marker genes (*RORA, CCL20*, and *IL23R)* of Cluster 2 (Th17) across the CAR and mCAR groups in single-cell RNA sequencing; **E** Dot plots depict the single-cell expression of the selected genes of Cluster 2 (Th17) across these two groups. CD19 CAR T-cells were co-cultured with Raji cells at a E: T ratio of 1: 5 in the presence or absence of hUC-MSCs. **F** Cytokine concentrations (IL-1β, IL-6, TGF-β, and IL-17A) in the supernatant were measured at 48 h, with levels in the CAR T only control group falling below the detection limit (*n* = 3); **G** Exogenous IL-1, IL-6, anti-IL1β and anti-IL6 were added to the co-culture systems, and specific lysis was tested at 48 h (n = 3). Two-sided unpaired *t*-test were used to assess significance in (**F**). One-way ANOVA was used in (**G**). *p < 0.05; **p < 0.01; ***p < 0.001.

To further explore the mechanisms by which MSCs mediate the NK-like CD8^+^ CTL phenotype, we performed pathway enrichment analysis on the bulk RNA sequencing profile of CAR T-cells. The analysis revealed that the mCAR-T group showed enhanced enrichment of IL-17 and Th17-related signaling pathways in KEGG, GO (Fig. 4A, B), and GSEA analyses (Fig. 4C). We then compared the characteristics of Cluster 2 between the CAR and mCAR groups in single-cell transcriptome, which defined as a Th17 subset. We found no notable differences in the expression of highly-expressed markers (*RORA*, *CCL20*, and *IL23R*) associated with Th17 cells (Fig. 4D). However, we observed increased activation (*ICOS*, *ZAP70*, *CD69*, *IL2RA*, *TNFSF8*, and *IL2*) and effector (*IL15*, *KLRD1*, *KLRC1*, *KLRC3*, *IFN*, and *PRF1*) markers, and decreased exhaustion (*LAG3*, and *PDCD1*) markers of Th17 cells in the dual-cell immunotherapy group (Fig. 4E), which indicating a more fit subset of Th17 cells. These findings indicate that the enhanced anti-tumor activity of mCAR T-cells may be associated with the IL-17/Th17 pathways.

Studies demonstrate that low-concentration TGF-β synergizes with IL-6 to drive STAT3-dependent differentiation of naïve CD4^+^ T cells into Th17 cells, while IL-1β potentiates Th17 polarization [38]. IL-17A is the main effector molecule of Th17 cells [39]. Subsequently, we examined the upstream factors (IL-6, IL-1β, and TGF-β) and the downstream factor (IL-17A) associated with the Th17 differentiation pathway. Strikingly, in the in vitro cytotoxicity assay, the mCAR-T group exhibited significantly higher levels of these cytokines compared to the CAR-T group (Fig. 4F). We next conducted in vitro co-culture killing assays with the addition of cytokines, IL-1β or IL-6, or antibodies against IL-1β or IL-6, and the cytokine concentrations were based on those measured in the co-culture supernatants. The addition of IL-1β did not enhance CD19 CAR T cell-mediated lysis in both the CAR-T and mCAR-T groups, while the addition of IL-6 decreased CD19 CAR T cell-mediated lysis. Conversely, addition of anti-IL-1β, anti-IL-6 or both led to an increase in CD19 CAR T-cell cytolytic ability (Fig. 4G). These data suggest that IL-1β and IL-6 do not exert direct effects on the enhancement of CAR T-cell efficacy, but rather, may act as inhibitory factors.

### Dual cellular immunotherapy boosts CD8^+^ CAR-T cytotoxicity via Th17 pathway for enhanced antitumor efficacy

**Fig. 5:**
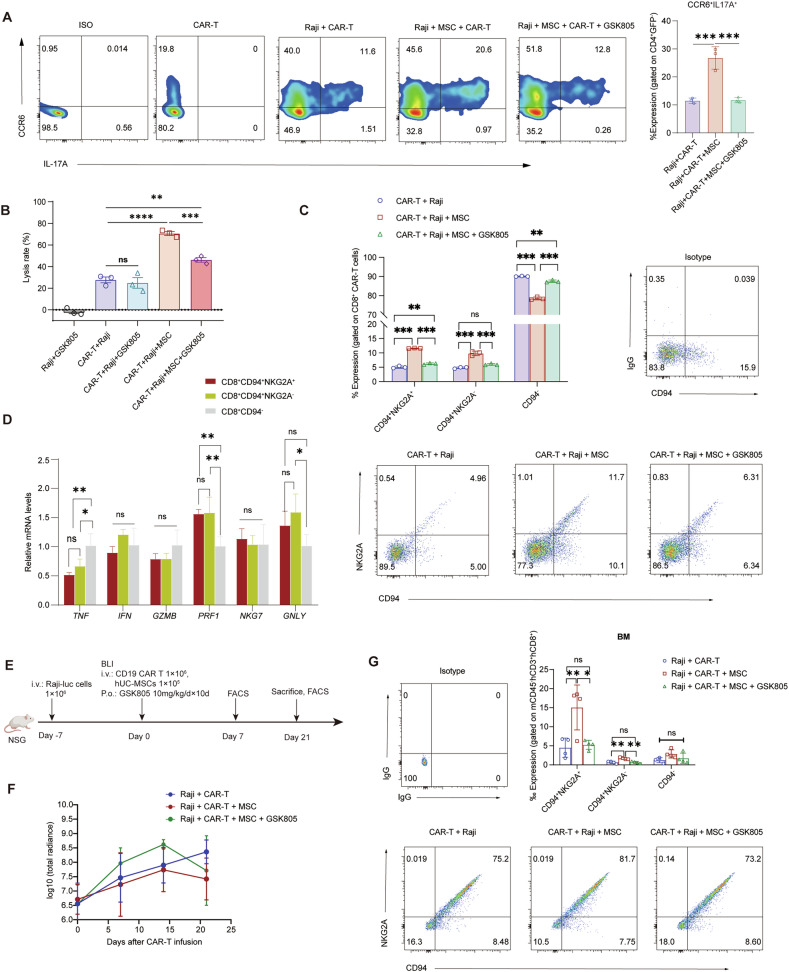
Dual cellular immunotherapy boosts CD8^+^ CAR T cytotoxicity via Th17 pathway for enhanced antitumor efficacy. CD19 CAR T-cells were co-cultured with Raji cells at a E: T ratio of 1: 5, in the presence or not of hUC-MSCs. CAR T-cells were collected for assessment by flow cytometry at 48 h; **A** Flow cytometry analysis of CD4^+^IL17A^+^CCR6^+^ cells, demonstrating a significant increase in the Th17 cell subset in the mCAR group, with subsequent downregulation after RORγt inhibition with GSK805. Data are representative of at least three independent experiments (*n* = 3); **B** Increase of CD19 CAR T-cell cytolysis by MSCs was partially reversed by GSK805. (*n* = 3); **C** MSC-upregulated CD94 and NKG2A expression in CD8^+^ CAR T-cells is reversed by the Th17 differentiation inhibitor GSK805. Bar graph shows the proportional distribution of CD8⁺CD94⁺NKG2A⁺, CD8⁺CD94⁺NKG2A⁻, and CD8⁺CD94⁻ subpopulations within CD8⁺ CAR T-cells across experimental groups. Scatter plots represent schematic distributions of individual data points. **D** CD19 CAR T-cells were co-cultured with Raji cells at a E: T ratio of 1: 5 in 6-well plates for 48 h. Live cells (GFP⁻FVD⁻) were sorted into three distinct populations based on CD8, CD94, and NKG2A expression: CD8⁺CD94⁺NKG2A⁺, CD8⁺CD94⁺NKG2A⁻, and CD8⁺CD94⁻. RNA was extracted from each population, reverse transcribed into cDNA, and subjected to quantitative PCR (qPCR) analysis to determine the relative mRNA expression levels of TNF, IFN-γ, GZMB, PRF1, NKG7, and GNLY. **E** Experimental design of the in vivo experiment on the antitumor effect of MSC on CD19 CAR T-cells, which were purified by sorting after lentiviral transfection and expansion in vitro for 7 days before being i.v. injection into mice. The Th17 differentiation inhibitor GSK805 was given by gavage at 10 mg/kg/day (*n* = 5); **F** Tumor growth monitored by BLI at the indicated time points (*n* = 4 or 5); **G** Flow cytometric analysis of human CD8⁺ T cell subsets in BM at day 7 post CAR-T infusion. MSC treatment increased CD8⁺CD94⁺ CAR T-cell frequencies in BM, consistent with in vitro findings. Data represent mean ± SD (*n* = 4). One-way ANOVA was used to assess significance in (**A–D**,**G**). *p < 0.05; **p < 0.01; ***p < 0.001.

Muranski et al. demonstrated that Th17 cells enhance the anti-tumor immune response of cytotoxic T-cells in a murine melanoma model, an effect dependent on IFN-γ signaling [40]. Based on the single-cell subpopulation marker analysis, we employed a combination of CD4, IL-17A, and CCR6 co-staining in our in vitro flow cytometry experiments to precisely identify Th17 cells. FCM analysis confirmed that mCAR-T group significantly promoted Th17 cell differentiation. (Fig. 5A). GSK805, a retinoic acid receptor-related orphan receptor gamma (RORγ) antagonist, has been shown to effectively suppress Th17 differentiation. Upon GSK805 addition, the upregulation of IL-17A in mCAR-T group was significantly inhibited (Fig. 5A). Moreover, it partially reversed the enhanced anti-tumor efficacy (Fig. 5B). Within CD8^+^ T cells, the CD94^+^ (*KLRD1* encoded) subset was identified as NK-like CTLs [41]. CD8^+^ T cells in the mCAR-T group showed markedly increased expression of CD94 compared to the CAR-T group. The function of CD94 is determined by its pairing with NKG2 family members, forming either an inhibitory complex with NKG2A or an activating complex with NKG2C/E [41]. We found that hUC-MSCs upregulated both CD94^+^NKG2A^+^ and CD94^+^NKG2A^-^ populations within CD8^+^ CAR T-cells, an effect that could be reversed upon inhibition of Th17 differentiation with GSK805 (Fig. 5C). To assess the function of these subsets, we sorted CD8^+^ CAR T-cells into three distinct populations post-stimulation. qPCR analysis revealed a graded increase in the expression of cytotoxic molecules *GNLY* and *PRF1*, following the order: CD94^+^NKG2A^-^ > CD94^+^NKG2A^+^ > CD94^-^ (Fig. 5D). Collectively, these data indicate that hUC-MSCs promote the expansion of a highly cytotoxic, CD94^+^CD8^+^ CAR T-cell subset via a mechanism associated with Th17 differentiation.

To investigate whether hUC-MSCs exert a similar regular effect on CD19 CAR T-cell differentiation in vivo, we used an NSG humanized mice model of NHL. Inhibition of Th17 cell differentiation in vivo was achieved by giving 10 mg/kg daily of GSK805 to mice from the day of CD19 CAR T-cells (1 × 0^6^ cells) and hUC-MSCs (1 × 10^5^ cells) administration and continued for 10 days (Fig. 5E). We observed a trend in increased tumor growth in the GSK805 group compared to hUC-MSCs or CAR T-cell alone groups (Fig. 5F). However, no significant difference in short-term survival was observed among the three groups (Fig. S5A). Based on previous experience with CAR T-cell expansion assay in PB, we selected day 21, the peak expansion time, as the detection time point. Neither hUC-MSCs nor GSK805 significantly affected CAR T-cell expansion (Fig. S5B). Analysis of BM from tumor-bearing mice revealed that MSCs co-administration significantly increased the frequencies of both CD8⁺CD94⁺NKG2A⁺ and CD8⁺CD94⁺NKG2A⁻ CAR T-cell subsets compared to controls (Fig. 5G). This expansion was suppressed by GSK805 treatment, which specifically reduced the proportions of these CD94⁺ populations without affecting the CD94⁻ counterpart. No significant differences in these subsets were detected in the spleen or peripheral blood (Fig. S5C). Together, these findings indicate that hUC-MSCs enhance CD8⁺ CAR T-cell cytotoxicity in vivo by expanding the CD94⁺ subset through a Th17-dependent mechanism.

### Dual cellular immunotherapy mitigates CRS while potentiating anti-tumor activity

**Fig. 6:**
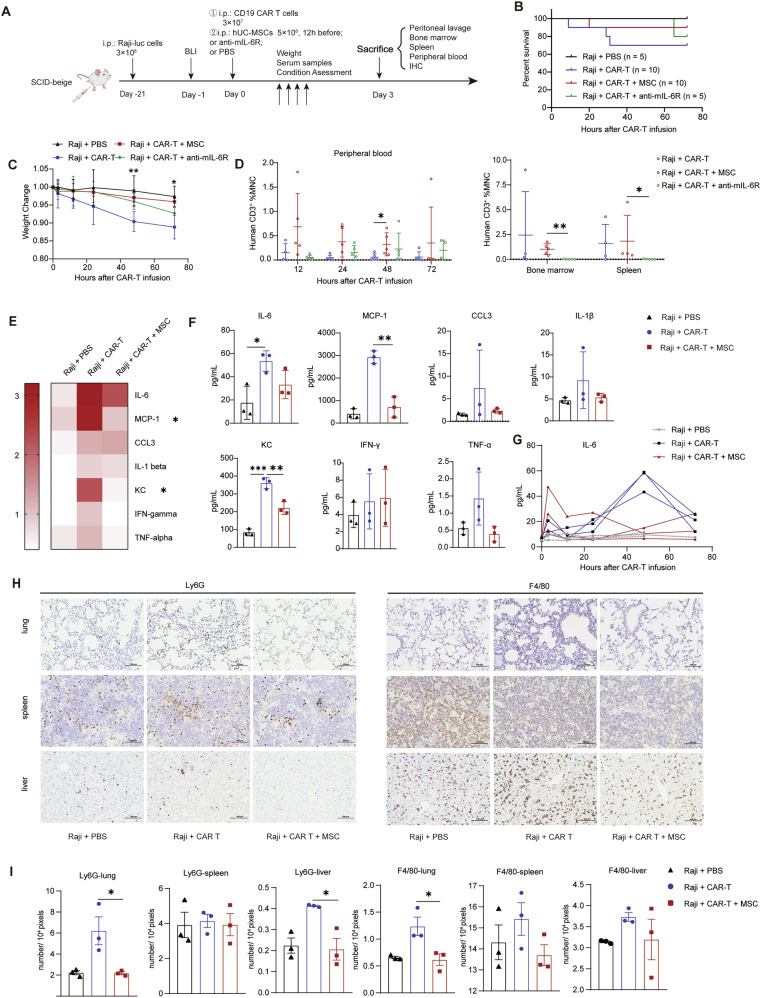
Dual cellular immunotherapy mitigates CRS. **A** Schematic drawing showing the experimental design: 3 × 10⁶ Raji-Luc cells were injected intraperitoneally (i.p.) into SCID-beige mice. Tumor growth was tracked via bioluminescence, and 3 × 10⁷ CD19 CAR T-cells were administered with or without 3 × 10⁶ hUC-MSCs intraperitoneally 12 hours prior to treatment; **B** Kaplan-Meier survival analysis demonstrates prolonged survival in the MSC pre-treated groups (≥ 4 independent experiments); **C** Weight change of mice following transfer of CAR T-cells, normalized to pre-transfer weight (*n* = 5 or 6); **D** CAR T-cell expansion in PB, bone marrow, and spleen at the indicated time points (*n* = 5). The MSC group exhibited better amplification compared to mice treated with anti-mIL-6R and untreated; **E**,**F** Mouse cytokine/chemokine heatmap and histogram (serum collected 48 h after CAR T-cell injection) exhibits increased cytokine and chemokine levels, typical of CRS. hUC-MSCs resulted in a significant decrease in several cytokines and chemokines compared with the CAR group. The fold change was normalized to serum collected before CAR T-cell transfer (*n* = 3); **G** Serum samples were collected from CRS model mice at indicated timepoints (pre-infusion, 3, 12, 24, 48, and 72 h after CAR-T infusion). Cytokine concentrations were quantified using Bio-Plex Pro Mouse Cytokine Assays (Bio-Rad, CA) following manufacturer’s protocols. **H**,**I**. Immunohistochemical staining of Ly6G and F4/80 expression in lung, spleen, and liver tissues 72 h after CAR T transfer. Representative images from three mice per group are shown; the positive cell density from three random images per sample was statistically analyzed (*n* = 3). One-way ANOVA was used to assess significance in (**D–F**,**H**). *p < 0.05; **p < 0.01; ***p < 0.001.

CRS is a common and potentially life-threatening complication following CAR T-cell therapy, characterized by a massive cytokine surge (including TNF-α, IL-6, and IL-1β), which triggers a severe systemic inflammatory response [42]. We investigated the role of hUC-MSCs in a CRS model in SCID-beige mice with intact macrophage function, as shown in the flowchart in Fig. 6A [27]. Mice with Raji cell-based high tumor burden (3 × 10^6^ cells) rapidly experienced weight loss or even death after receiving high doses of CAR T-cells (3 × 10^7^ cells). IL-6R inhibitors, the standard treatment for CRS, improved the survival rate and alleviated weight loss symptoms, indicating that our CRS model mimics the patients’ response (Fig. 6B, C). Administering hUC-MSCs (5 × 10^6^ cells) 12 hours *prior* to CD19 CAR T-cell infusion led to reduced mortality and significantly less weight loss, with statistically significant differences at 48 and 72 hours (Fig. 6C). Flow cytometry analysis further showed that proliferation of T-cells significantly increased in the spleen, BM, and PB in the mCAR-T group compared to the anti-mIL-6R group (Fig. 6D).

IL-6, primarily produced by monocytes and macrophages, plays a crucial role in the pathogenesis of CRS [43]. MCP-1 (CCL2) is also critical for inflammation, while KC (CXCL1) attracts myeloid cells, including neutrophils. A rapid increase in these CRS-linked cytokines was observed post CAR T-cell infusion, as measured by Luminex. The hUC-MSC-treated group exhibited significant downregulation of MCP-1 and KC, while showing a decreasing trend (though not statistically significant) in IL-6, IL-1β, CCL13, and TNF levels (Fig. 6E, F). Kinetic analysis of IL-6 in the CRS mouse model revealed distinct temporal patterns between treatment groups. In mCAR-T-treated mice, serum IL-6 levels peaked during the early phase (within 12 hours post-infusion) and subsequently declined to baseline. In contrast, mice receiving CAR T-cells alone exhibited a delayed IL-6 peak, reaching maximal levels approximately 2 days after infusion (Fig. 6G). Thus, hUC-MSCs mitigate CRS severity without affecting T-cell expansion in vivo.

Excessive cytokine release from macrophages, following CRS onset, increases vascular permeability and induces endothelial cell damage. Concurrently, chemokines recruit a substantial influx of neutrophils to the site of injury, contributing to multiple organ dysfunction [44]. To investigate the effect of hUC-MSC on the inflammatory response following CRS, the composition of immune cell infiltration was analyzed. Immunohistochemical analysis revealed significantly reduced neutrophil infiltration in both lungs and liver, along with decreased macrophage infiltration specifically in the lungs of mCAR-T treated mice (Fig. 6H, I). While the spleen and liver showed non-significant decreasing trends in macrophage infiltration. This suggests that hUC-MSCs mitigate organ inflammation caused by CRS, reducing an overactive immune microenvironment, which may prevent organ failure.

### Dual cellular immunotherapy mitigates CRS by downregulating hyperactivated macrophages and promoting an antitumor microenvironment

**Fig. 7:**
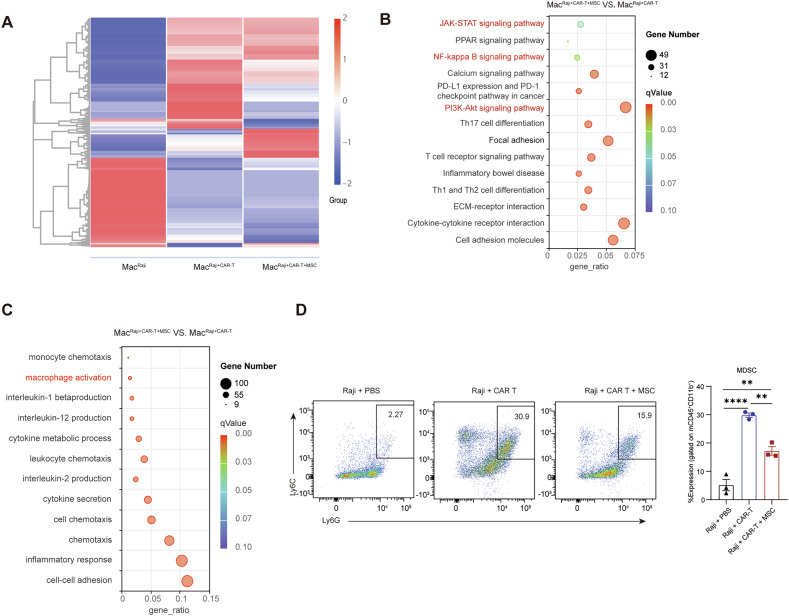
Dual cellular immunotherapy mitigates CRS by regulating macrophage activation and creating a more favorable tumor microenvironment. Monocytes were collected from peritoneal lavage 72 h post-CAR T transfer as shown in Fig. 6A, and macrophages were sorted for RNA-seq or analyzed by FCM using the gate of CD45^+^CD11b^+^; **A** Hierarchical clustering of RNA-seq data shows significantly different gene expression profiles in macrophages from different groups (Mac^Raji^: Raji + PBS group; Mac^Raji+CAR T^: Raji + CD19 CAR T group; Mac^Raji+CAR T+MSC^: Raji + CD19 CAR T + MSC group) (*n* = 1); **B**,**C** KEGG and GO clustering of genes significantly downregulated in Mac^Raji+CAR T+MSC^ versus Mac^Raji+CAR T^; **D** FCM plot and histogram showing MDSCs (Ly6C^high^Ly6G^high^) gated on CD45^+^CD11b^+^ (*n* = 3). One-way ANOVA was used to assess significance in (**D**). **p < 0.01; ***p < 0.001.

In CRS, macrophages are activated by various stimuli, including tumor cells, immunotherapeutic cells, and damage-associated molecular patterns, which drive their polarization toward a pro-inflammatory state and amplify the inflammatory response [45]. To examine the impact of hUC-MSCs on macrophage function, RNA sequencing was conducted on mouse F4/80⁺CD11b⁺ cells isolated from the peritoneum 48 hours after CD19 CAR T-cell infusion (Fig. 6A). Transcriptional analysis revealed a distinct profile of differentially expressed genes in macrophages in the hUC-MSCs pre-treated group (Fig. 7A). KEGG enrichment analysis revealed downregulation of inflammation-related pathways, including NF-kappa B, JAK-STAT, cytokine response, and cell adhesion pathways (Fig. 7B). GO enrichment analysis also showed downregulation of cytokine-related pathways, such as IL-1β and IL-2, and of processes like neutrophil migration and chemotaxis, most prominent for macrophage activation (Fig. 7C). These findings align with the observed reduction in cytokine levels and a trend toward a decreased presence of peritoneal neutrophils and monocytes in the hUC-MSC-treated group (Fig. S6A).

Suppressive immune cells, such as myeloid-derived suppressor cells (MDSCs), tend to increase in states of high tumor burden. To determine whether hUC-MSCs promote a favorable TME while reducing inflammation, MDSCs (Ly6C^high^Ly6G^high^) from peritoneal lavages were evaluated. A significantly lower percentage of MDSCs was observed in the hUC-MSCs pre-treated group (Fig. 7D). Collectively, these data show that hUC-MSCs can regulate macrophage polarization without inducing a suppressive TME.

## Discussion

In the evolving field of CAR T-cell therapy, addressing T cell dysfunction and safety concerns, particularly under conditions of high tumor burden, remains a critical challenge. This study explores the efficacy and safety of a dual cellular combination therapy combining hUC-MSCs with CD19 CAR T-cells in the context of high tumor burden in lymphoma. The findings underscore the potential of this strategy for enhancing anti-tumor activity while optimizing immune cell function.

The combination of MSCs with CAR T-cells has been the subject of considerable debate, primarily because of concerns that it may compromise anti-tumor efficacy. This is largely in reference to the well-documented immunosuppressive properties of MSCs, which broadly inhibit T cell proliferation and cytokine secretion [46]. We did observe that hUC-MSCs inhibited the expansion of anti-CD3/28 stimulated CAR T-cells and the secretion of some cytokines (IL-2, TNF, and IFN-γ) (data not show). No significant effect of MSCs on CAR T-cells was observed at a higher E:T ratio in in vitro experiments. There was also no significant difference in survival and tumor killing between the CAR-T and mCAR-T groups in a relatively low burden *vivo* model. These results align with previous reports [18, 19]. However, enhanced anti-tumor efficacy was observed at a lower E:T ratio in vitro, once-and continual-killing assay; in vivo, the combination therapy also prolonged the survival of mice with high tumor burden. Thus, enhanced CAR T-cell activation is prominent under conditions of high tumor burden, as validated by transcriptomic data. In addition, the dose of MSCs may critically influence the outcomes of dual immunotherapy. Currently, limited data are available on dosage optimization of MSCs in combination with CAR T-cell therapy, as the field remains exploratory. In two previously reported humanized mouse tumor models utilizing combination therapy, MSCs were administered at 5 × 10⁶ cells per dose [19, 47], which resulted in a modest and not significant reduction in early tumor killing compared to CD19 CAR T-monotherapy. Notably, our study employed a lower hUC-MSCs dose (5 × 10^5^ cells) and yet achieved better outcomes in the mouse model, suggesting a potential MSC dose-dependent therapeutic window. This warrants further investigation to define the optimal parameters for synergistic efficacy. Beyond dosage, administration timing represents another critical parameter. Our findings demonstrate that MSC infusion within 24 h post-CAR T achieves optimal efficacy-safety balance, enabling early immunomodulation before inflammatory escalation while preserving CAR-T functionality.

Our study is the first to use single-cell sequencing to elucidate the impact of MSCs on CAR T-cell phenotype and function. Single-cell transcriptomic analysis showed an enhanced effect of the CD8^+^ T cell population in the mCAR-T group, due to MSC-mediated induction of Th17 differentiation. The role of Th17 cells in cancer remains controversial; however, accumulating evidence indicates that they can potentiate long-term anti-tumor immunity through multiple pathways [40, 48, 49]. In our study, the upstream molecules inducing Th17 cell differentiation, e.g., IL-6 and IL-1β [50], were increased at both the transcriptomic and protein levels. Enrichment analysis validated the differentiation of Th17 cells, and when GSK805, known to inhibit transcription factor RORγt, blocked the differentiation of Th17 cells, were added, their antitumor effect was reversed in vitro and in vivo, highlighting the importance of the Th17 subset in MSC-mediated enhancement of CAR T-cell activity. Using single-cell RNA sequencing analysis, Cluster 0, defining Th17 cells, exhibited higher activation in the mCAR-T group, with significant downregulation of exhaustion markers, *HAVCR, PDCD1*, and *LAG3*. These results indicate that hUC-MSCs can enhance CAR T-cell activation without increasing exhaustion, a significant issue in CAR T-cell therapy.

The composition and phenotype of CAR T-cells influence their function, and single-cell sequencing technology has improved the decoding of CAR T-cell composition and phenotype [51]. An optimal proportion of CD4^+^ T cells can enhance the long-term efficacy of the CAR T-cells [36]. Tex adversely affects the persistence and expansion of CAR T-cells, whereas Tcm are associated with lower relapse rates [52]. In our study, in vitro functional assays demonstrated no significant difference in exhaustion markers (PD-1, LAG3, and TIM3) between the CAR-T and mCAR-T groups. Single-cell RNA sequencing analysis from in vitro co-cultures showed that the mCAR-T group had a comparable CD4/CD8 ratio and increased CD4 Tcm (Cluster 1), but increased PDCD1^+^ Tex population. The increased CD4 Tcm in the mCAR-T group supports the superior expansion ability of CAR T-cells in mice.

Notably, NK-like CTLs (Cluster 0) with higher effector and activation gene expression, but lower exhaustion gene expression, were dramatically increased in the mCAR-T group. Mechanistically, these NK-like CTLs are primarily composed of CD8⁺CD94⁺ subsets, which exhibit elevated expression of key cytotoxic effectors, including perforin and granzymes, thereby providing a molecular basis for their superior tumor-killing capacity. Previous research indicated that cytotoxic T lymphocytes exhibit significant plasticity, converting into NK-like cells [37, 53]. This manifests by the expression of cytotoxic proteins such as perforin and granzymes in CD8^+^ CTLs [54], and that of the NK receptor *NKG2C*, which mediates the TCR-dependent or -independent antimicrobial activity [55, 56]. This conversion process is not entirely beneficial. Good et al. found that NK-like CTLs can be segregated into two distinct clusters: dysfunctional and non-dysfunctional [37]. The dysfunctional clusters, characterized by high expression of exhaustion-related phenotypic molecules such as *HAVCR2, PDCD1*, and *LAG3*, are the key culprits in the failure of CAR T-cell therapy. For example, June et al. reported that, in pancreatic cancer, mesothelin-redirected CAR T-cell dysfunction is associated with the transition of CD8^+^ T cells into NK-like CTLs, highly expressing exhaustion characteristics [37]. To address this challenge, researchers have attempted to restore CAR T-cell function by knocking out exhaustion-related genes using gene-editing technology or blocking exhaustion pathways with immune checkpoint inhibitors [57–59]. Although these methods have achieved some success, the complexity of the TME renders single-target interventions often unable to comprehensively address the coexistence of multiple exhaustion mechanisms. Through single-cell subpopulation analysis, in vitro models clearly showed the transition of NK-like CTL in mCAR-T group. Surprisingly, we found that the transformed NK-like CTLs enriched in mCAR-T group and characterized with comparatively low exhaustion scores, even though enrichment analysis of this cell population revealed downregulation of the naïve pathway. The unique transcriptional profile of NK-like CTLs suggests that MSCs may enhance CAR T-cell efficacy, while preventing their exhaustion, which may underlie the superior outcomes observed with the hUC-MSC + CD19 CAR T combination therapy. Th17 cells are known to augment the cytotoxic activity of CD8^+^ T- cells [40]. In the in vitro co-cultured assay, the CD8^+^CD94^+^ T-cells were supposed to represent NK-like CTLs, with their percentage significantly decreased with the inhibition of Th17 differentiation. Thus, the expansion of NK-like CTLs is associated with Th17 differentiation.

Emerging concerns about the side effects of CAR T therapy, including the association of CAR T-cell expansion with severe CRS, are getting increasing attention [43]. Treating CRS requires a balance between mitigating adverse effects and preserving the efficacy of CAR T-cell therapy. Clinical trials involving early administration of tocilizumab have shown mixed results, with improvement in CRS severity but heightened neurotoxicity due to increased IL-6 levels [60]. Our study demonstrates that hUC-MSCs provide broader cytokine suppression during CRS compared to current clinical approaches. Importantly, the timing of MSC administration is crucial for achieving this balance. Administering MSCs within the 24 h after CAR-T infusion effectively suppresses the onset of late-stage cytokine storms while preserving the early IL-6 signaling that promotes Th17 differentiation and sustains CAR-T anti-tumor efficacy. MSCs have been shown to suppress inflammatory cytokines like IL-6 and IL-1, in various models of sepsis, peritonitis, and severe COVID-19 [61–63]. Their role in protecting organs from CRS supports our findings that hUC-MSCs can reduce CRS-induced inflammation and potentially prevent related organ failure, a benefit not currently achievable with existing cytokine inhibitors. However, the reduction of MDSCs observed in our CRS model contrasts with previous reports describing MSC-induced MDSC expansion, highlighting the context-dependent nature of MSC-mediated immunomodulation and the need for further investigation.

In summary, our study reveals that hUC-MSCs enhance the anti-tumor function of CAR T-cells by inducing Th17 cell differentiation to augment CD8+ T cell activity under high tumor burden conditions, while alleviating CRS by downregulating hyperactivated macrophages. These findings suggest that hUC-MSCs could offer dual benefit through improving CAR T-cell therapy efficacy and safety. Thus, our study provides a strong premise for further evaluation of the safety profile and clinical efficacy of this dual cellular immunotherapy strategy. Future research should inquire further into the mechanisms underlying the effects of dual cellular combination therapy and explore its potential in other tumor types, offering hope to a broader patient population.

## Supplementary information

Supplemental material

## Data Availability

Sequencing data have been deposited at the National Omics Data Encyclopedia (NODE, https://www.biosino.org/node/) under accession number OEP00006655. Data that support this study are available upon request from the corresponding author, Ting Yang (yang.hopeting@gmail.com).
