# Supplementary material for: Th17-driven CD8+ T cells in hUC-MSC and CAR T-cell dual immunotherapy for superior anti-tumor efficacy
Source: Cell Death Dis. 2026 Mar 27;17(1):418. doi: 10.1038/s41419-026-08656-7 (PMC13149661; doi:10.1038/s41419-026-08656-7)
Supplement: Supplementary file 1 — Supplemental material [file 41419_2026_8656_MOESM1_ESM.docx]

**Supplementary Material**

**FIGURE**

**Figure S1**

**
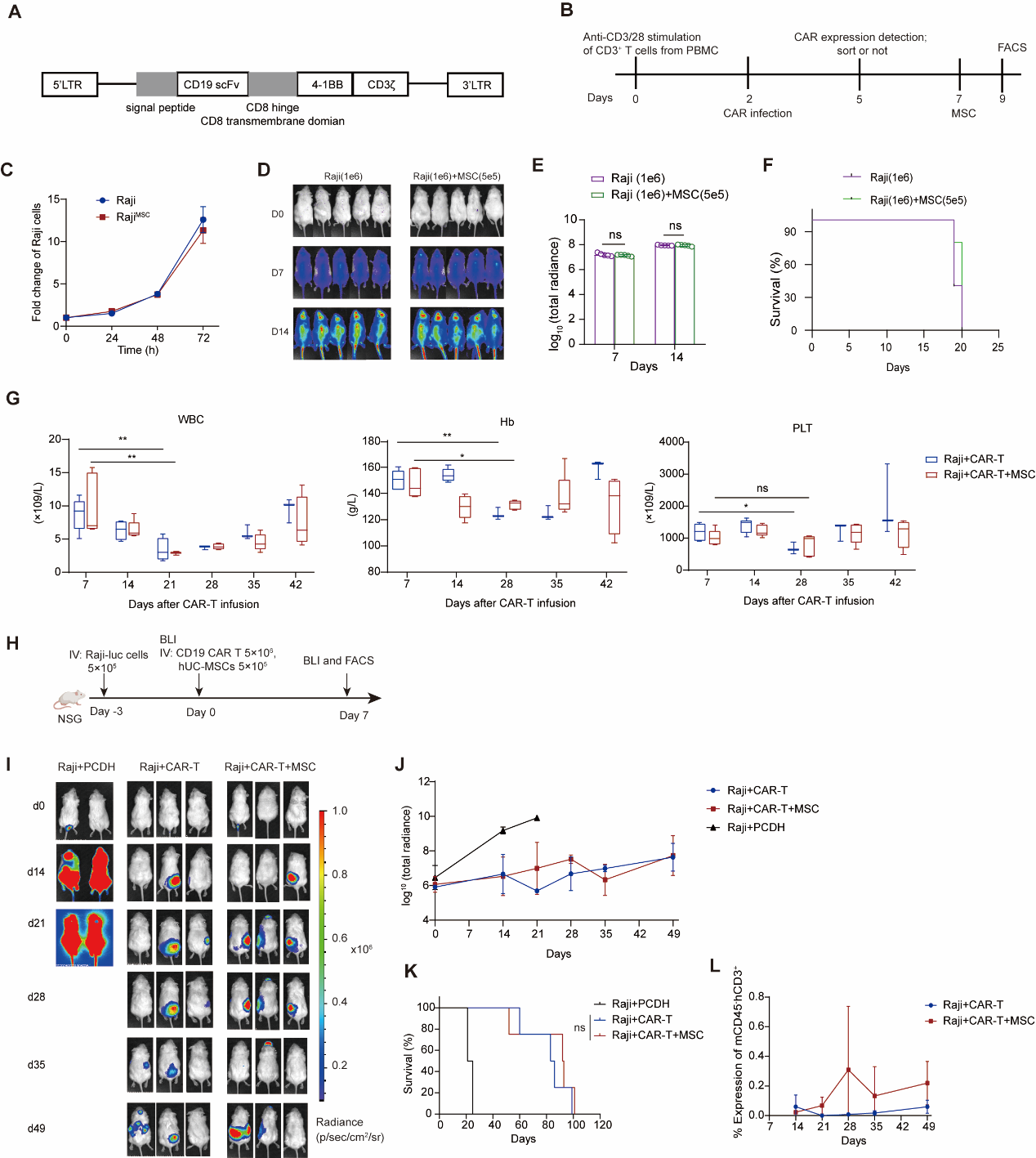
**

**Figure S1** **Experimental design and evaluation of the effects of hUC-MSCs on CD19 CAR-T cell function *in vitro* and *in vivo***. **(A)** Diagram illustrating the construction of the CD19 CAR sequences; **(B)** Flowchart of CD19 CAR T-cells culture and functional tests; **(C)** Continuous monitoring of Raji proliferation when co-cultured with hUC-MSCs at a 2:1 ratio (n = 3); **(D-F)** The effects of hUC-MSCs on *in vivo* expansion of tail vein-injected Raji cells in NSG mice, and on mice survival (n = 5). Raji cells (1×10^6^), with or without hUC-MSC (5×10^5^), were intravenously injected into NSG mice to assess tumor growth and survival *in vivo.* **(G)** Blood counts were evaluated with the Sysmex flagship analyzer at the indicated time points (WBC: white blood cell, Hb: hemoglobin, PLT: platelet); **(H)** Flowchart of the *in vivo* experiments design evaluating the impact of hUC-MSC on the efficacy of CD19 CAR-T therapy in low tumor burden; **(I-J)** Tumor growth monitored by bioluminescence (BLI) at the indicated time points; **(K)** Kaplan-Meier survival analysis shows no significant survival benefit for NSG treated with CAR T-cells with hUC-MSCs compared to the control group (n = 3 for each group); **(L)** CD19 CAR T-cell expansion in peripheral blood at the indicated times. Statistical significance was assessed using the log-rank test (**K**) and two-tailed unpaired *t*-test (**C-J** and **L**). **p* < 0.05; ***p* < 0.01; ****p* < 0.001.

**Figure S2**

**
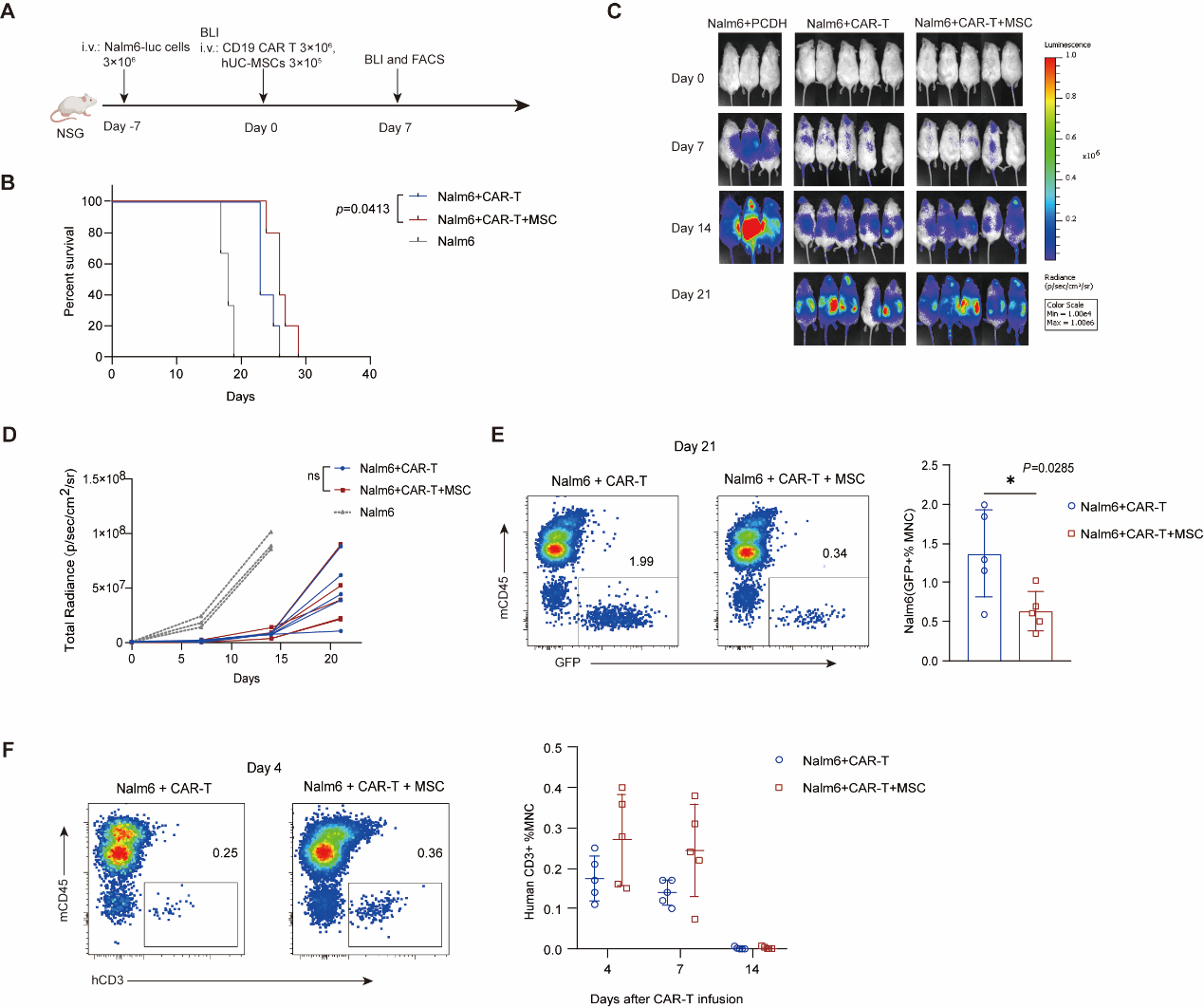
**

**Figure S2 Impact of hUC-MSCs on the anti-tumor efficacy of CD19 CAR-T cells against Nalm6 leukemia *in vivo*. (A)** Schematic diagram of the experimental animal design and treatment timeline. **(B)** Kaplan-Meier survival curves of mice in different treatment groups. **(C)** *In vivo* bioluminescence (BLI) imaging of tumor burden at the indicated time points. **(D)** Quantification of tumor burden over time. **(E)** Proportion of GFP⁺ tumor cells within peripheral blood mononuclear cells (PBMCs) at day 21, as assessed by flow cytometry. **(F)** Frequency of CAR-T cells within PBMCs at the indicated time points, measured by flow cytometry. Data are representative of two independent experiments (n = 5 mice per group). Statistical significance was assessed using the log-rank test (B) and two-way ANOVA (D-F). **p* < 0.05.

**Figure S3**

**
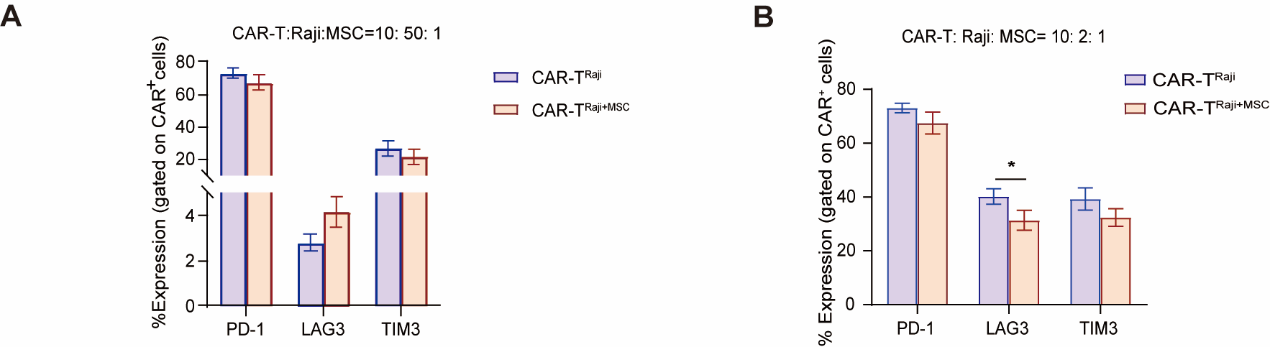
**

**Figure S3** **Analysis of the CAR-T cell exhaustion phenotype co-cultured with hUC-MSCs at 48 hours (n = 3).** **(A)** Exhaustion-related immunophenotype (PD-1, LAG-3, and TIM-3) of CAR-T cells with or without hUC-MSCs at a ratio of E: T = 10: 50. **(B)** Exhaustion-related immunophenotype of CAR-T cells with or without hUC-MSCs at a ratio of E: T = 5: 1. Statistical significance between treatment groups was determined using a two-tailed unpaired *t*-test. **p* < 0.05.

**Figure S4**

**
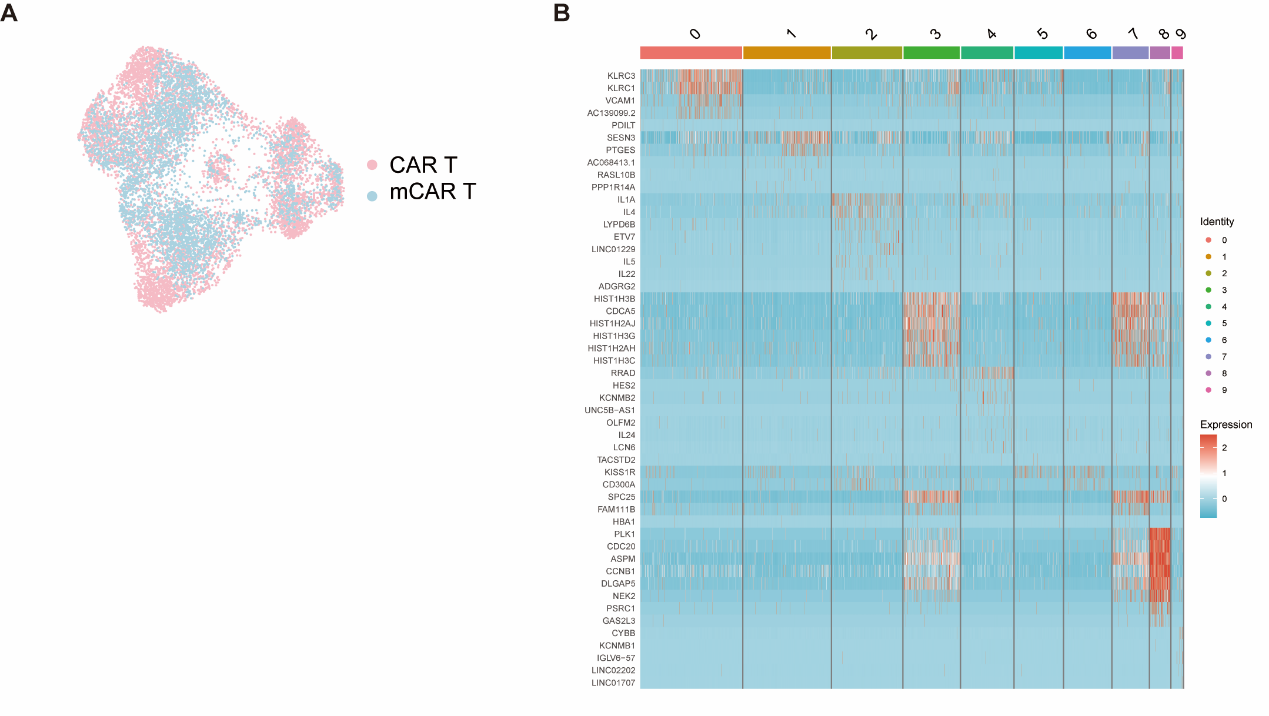
**

**Figure S4 Single-cell transcriptomic profiling reveals distinct clustering patterns and gene expression signatures between CAR-T and mCAR-T cells.** **(A)** UMAP visualization of all cells from CAR-T and mCAR-T groups; **(B)** Heatmap of differentially expressed genes, showing the top 10 genes for each cluster.

**Figure S5**


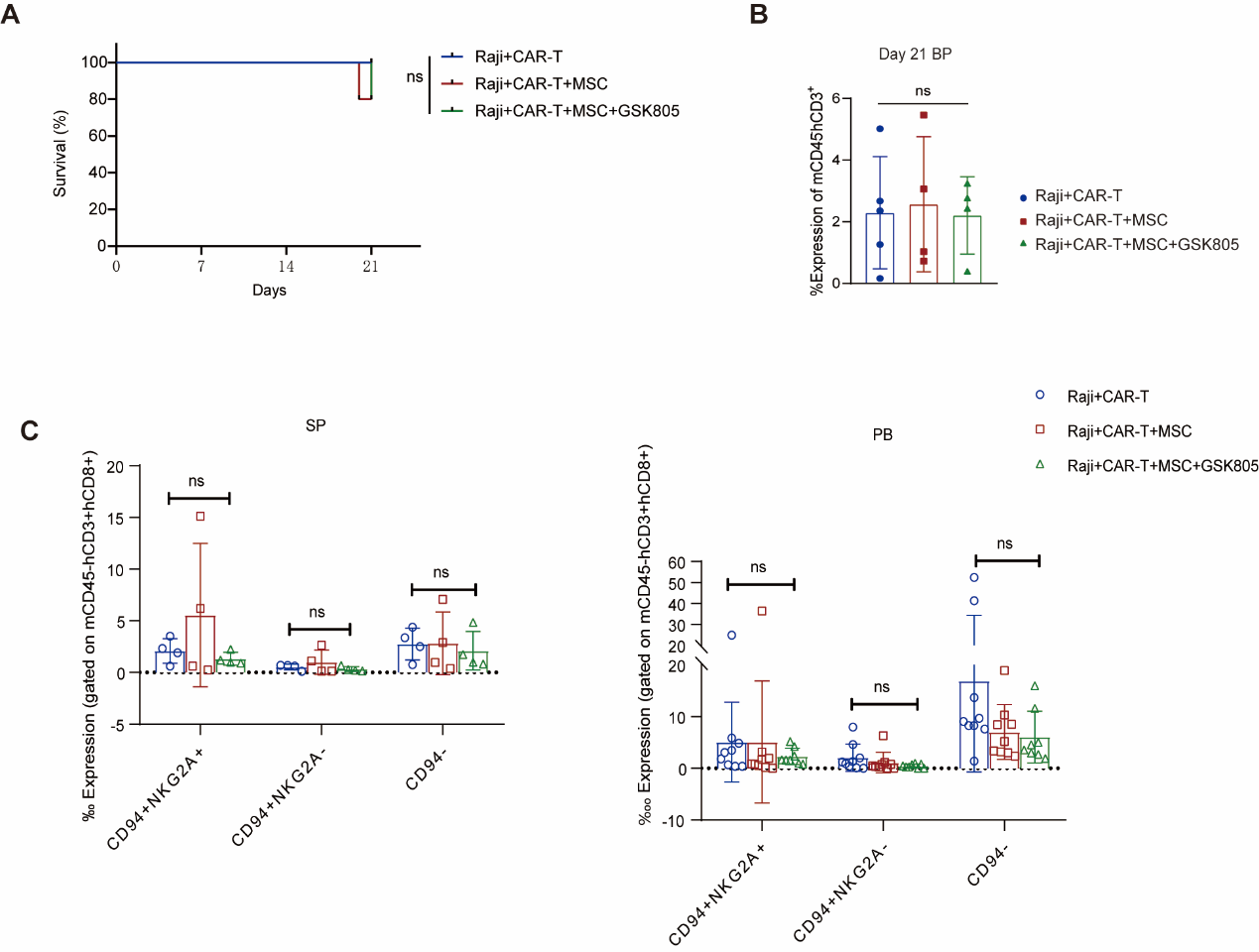


**Figure S5** **Th17 pathway inhibition does not enhance CAR-T cell efficacy *in vivo***. **(A)** Kaplan-Meier survival analysis shows that tumor-bearing NSG mice treated with hUC-MSCs and the Th17 differentiation inhibitor GSK805 at an early stage (n = 5); **(B)** CAR T-cell expansion in the peripheral blood at day 21, defined as human CD3^+^ mouse CD45^-^ cells; **(C)** Proportions of human CD8⁺CD94⁺NKG2A⁺, CD8⁺CD94⁺NKG2A⁻, and CD8⁺CD94⁻ subsets among mononuclear cells in spleen and peripheral blood at Day 7 (n = 4-9 for peripheral blood; n = 4 for spleen).

**Figure S6**


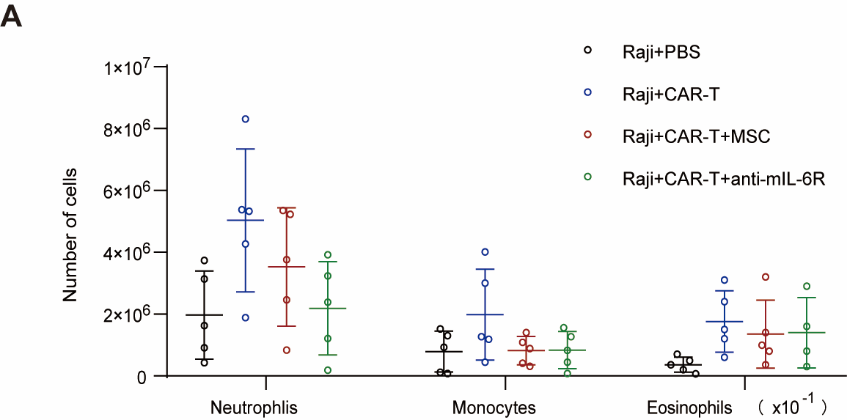


**Figure S6** **Peritoneal immune cell composition in CRS model mice following CAR-T cell infusion.** (A) Differential cell counts in peritoneal lavage fluid collected 72 hours after CAR-T cell infusion, following lavage with 5 mL PBS, and analyzed using an automated hematology analyzer (n = 5).

**Table S1. Antibodies used in this study**

| Antibodies | Source | Catalogue | | Clone | Application |
| --- | --- | --- | --- | --- | --- |
| PerCP/Cyanine5.5, anti-human CD3 | BioLegend | 300327 | HIT3a | | Flow cytometry |
| PE anti-human CD3 | BioLegend | 317307 | OKT3 | | Flow cytometry |
| APC anti-His Tag Antibody | BioLegend | 362605 | J095G46 | | Flow cytometry |
| APC/Cyanine7 anti-human CD25 | BioLegend | 302613 | BC96 | | Flow cytometry |
| PE anti-human CD69 | BioLegend | 388603 | FN50 | | Flow cytometry |
| PE/Cyanine7 anti-human CD107a | BioLegend | 328617 | H4A3 | | Flow cytometry |
| PE anti-human PD-1 | BioLegend | 379209 | A17188A | | Flow cytometry |
| APC/Cyanine7 anti-human TIM-3 | BioLegend | 345025 | F38-2E2 | | Flow cytometry |
| PE/Cyanine7 anti-human LAG-3 | BioLegend | 369309 | 11C3C65 | | Flow cytometry |
| PE anti-human IL-17A | BioLegend |  |  | | Flow cytometry |
| PE anti-human CD94 | BioLegend | 305506 | DX22 | | Flow cytometry |
| PE/Cyanine7 anti-human CD8 | BioLegend | 344711 | SK1 | | Flow cytometry |
| PerCP/Cyanine5.5 anti-human CD4 | BioLegend | 317427 | OKT4 | | Flow cytometry |
| APC anti-human CD161 | BioLegend | 307511 | W18070C | | Flow cytometry |
| APC anti-human/mouse Granzyme B Recombinant Antibody | BioLegend | 372203 | 4S.B3 | | Flow cytometry |
| APC anti-human IFN-γ | BioLegend | 502511 | QA16A02 | | Flow cytometry |
| Purified anti-human IL-1β | BioLegend | 508201 | JK1B-1 | | Neutralization |
| Purified anti-human IL-6 | BioLegend | 501101 | MQ2-13A5 | | Neutralization |
| PE Mouse IgG1 | BioLegend | 400113 | MOPC-21 | | Flow cytometry |
| PE/Cyanine7 Mouse IgG1 | BioLegend | 400125 | MOPC-21 | | Flow cytometry |
| APC/Cyanine7 Mouse IgG1 | BioLegend | 400127 | MOPC-21 | | Flow cytometry |
| APC/Cyanine7 anti-mouse CD45 | BioLegend | 157617 | QA17A26 | | Flow cytometry |
| PE anti-mouse CD11b | BioLegend | 101207 | M1/70 | | Flow cytometry |
| APC anti-human CD196 (CCR6) | BioLegend | 353415 | G034E3 | | Flow cytometry |
| APC anti-human NKG2A | BioLegend | 375107 | S19004C | | Flow cytometry |
| PE/Cyanine7 anti-human CD94 | BioLegend | 305515 | DX22 | | Flow cytometry |
| APC anti-mouse F4/80 | BioLegend | 157305 | QA17A29 | | Flow cytometry |
| BV421 anti-mouse F4/80 | BioLegend | 123131 | BM8 | | Flow cytometry |
| PerCP/Cyanine5.5 anti-mouse Ly6G | BioLegend | 127615 | 1A8 | | Flow cytometry |
| PE/Cyanine7 anti-mouse CD206 | BioLegend | 141719 | C068C2 | | Flow cytometry |
| BV510 anti-mouse CD86 | BioLegend | 105039 | GL-1 | | Flow cytometry |
| APC anti-mouse Ly6C | BioLegend | 128015 | HK1.4 | | Flow cytometry |
| FITC anti-mouse Ly6G | BioLegend | 127605 | 1A8 | | Flow cytometry |
| Anti-mouse IL-6R | BioXcell | BE0047 | 15A7 | | Intraperitoneally |
| Anti-mouse Ly6G | Servicebio | GB11229 | Polyclonal | | IHC* |
| Anti-mouse F4/80 | Servicebio | GB113373 | Polyclonal | | IHC |

*IHC, Immunohistochemistry.

**Table S2. Primers used for Real-time RT-PCR**

| Gene | Forward primer sequence | Reverse primer sequence |
| --- | --- | --- |
| *IFNG* | AGAGTGTGGAGACCATCAAGG | GGACATTCAAGTCAGTTACCGAA |
| *TNF* | GCCCATGTTGTAGCAAACCC | TGAGGTACAGGCCCTCTGAT |
| *GZMB* | CCAGGGCAGATGCAGACTT | CTCGTATCAGGAAGCCACCG |
| *PRF1* | TGATGCCACCATTCCAGGAG | CAGAGACAGGGGGCACTTG |
| *NKG7* | TGGGGACATCATATCAGGCCA | AGAGATGGCTCAGCTCCTCG |
| *GNLY* | AGGCTCCCTGCCCATAAAAC | ACCTCAAGGCCTGGGTTG |
| *ACTB* | CACCATGGATGATGATATCGC | CATAGGAATCCTTCTGACCCA |
